# Advances in Intrathecal Nanoparticle Delivery: Targeting the Blood–Cerebrospinal Fluid Barrier for Enhanced CNS Drug Delivery

**DOI:** 10.3390/ph17081070

**Published:** 2024-08-15

**Authors:** Ahmad Khalid Madadi, Moon-Jun Sohn

**Affiliations:** 1 Department of Biomedical Science, Graduate School of Medicine, Inje University, 75, Bokji-ro, Busanjingu, Busan 47392, Republic of Korea; khalidmadadi@yahoo.com; 2 Department of Neurosurgery, Neuroscience & Radiosurgery Hybrid Research Center, Inje University Ilsan Paik Hospital, College of Medicine, Juhwa-ro 170, Ilsanseo-gu, Goyang City 10380, Republic of Korea

**Keywords:** blood–cerebrospinal fluid barrier, nanoparticles, targeted drug strategies, CNS penetration, pharmacokinetics

## Abstract

The blood–cerebrospinal fluid barrier (BCSFB) tightly regulates molecular exchanges between the bloodstream and cerebrospinal fluid (CSF), creating challenges for effective central nervous system (CNS) drug delivery. This review assesses intrathecal (IT) nanoparticle (NP) delivery systems that aim to enhance drug delivery by circumventing the BCSFB, complementing approaches that target the blood–brain barrier (BBB). Active pharmaceutical ingredients (APIs) face hurdles like restricted CNS distribution and rapid clearance, which diminish the efficacy of IT therapies. NPs can be engineered to extend drug circulation times, improve CNS penetration, and facilitate sustained release. This review discusses key pharmacokinetic (PK) parameters essential for the effectiveness of these systems. NPs can quickly traverse the subarachnoid space and remain within the leptomeninges for extended periods, often exceeding three weeks. Some designs enable deeper brain parenchyma penetration. Approximately 80% of NPs in the CSF are cleared through the perivascular glymphatic pathway, with microglia-mediated transport significantly contributing to their paravascular clearance. This review synthesizes recent progress in IT-NP delivery across the BCSFB, highlighting critical findings, ongoing challenges, and the therapeutic potential of surface modifications and targeted delivery strategies.

## 1. Introduction

Effective drug delivery to the central nervous system (CNS) remains a formidable challenge due to the presence of anatomical and physiological barriers, notably the blood–brain barrier (BBB) and the blood–cerebrospinal fluid barrier (BCSFB). These barriers play critical roles in maintaining CNS homeostasis by regulating the exchange of molecules between the bloodstream and the CNS [1]. However, their restrictive nature significantly hampers the delivery of therapeutic agents to target sites within the brain and spinal cord, limiting the efficacy of treatments for various CNS disorders [2].

Systemic administration of medication, whether enteral or parenteral, often faces limitations in CNS drug delivery, such as poor drug penetration, off-target exposure, and side effects [3,4]. The oral bioavailability of the drugs is less than 100% due to several factors, while intravenous administration achieves 100% bioavailability [5,6]. Generally, 2~5% of the administered drug reaches the CNS [7,8,9]. However, effective concentration in the CNS is influenced by several factors, and many therapeutic medications face challenges in reaching sufficient doses to achieve a therapeutic effect.

Intrathecal (IT) delivery, involving the direct administration of drugs into the cerebrospinal fluid (CSF), has emerged as a promising strategy to bypass the BBB and BCSFB [10]. This method allows for higher drug concentrations in the CNS and can potentially improve therapeutic outcomes [10]. Despite these advantages, IT delivery still faces challenges, including limited penetration and distribution into the CNS, difficulties with sustained drug release, and the rapid clearance of active pharmaceutical ingredients (APIs) from the CSF.

Nanoparticles (NPs) have gained significant attention in recent years for their potential to enhance drug delivery systems (DDSs). Engineered NPs can be designed to extend drug circulation times, improve penetration into the CNS, and enable the sustained release of therapeutic agents. These properties make NPs an attractive option for overcoming the limitations associated with IT drug delivery [4]. Focusing on the BCSFB, which regulates the exchange of molecules between the bloodstream and CSF, presents a critical opportunity to enhance CNS drug delivery. By targeting the BCSFB, it is possible to develop more effective IT-NP delivery systems to achieve better therapeutic outcomes [11].

Key unmet pharmacokinetic (PK) needs for IT drug delivery include improving CSF retention, enhancing parenchymal penetration, achieving uniform distribution, and overcoming rapid clearance. IT-NPs show promise in addressing these issues [4]. Recent advances highlight the effectiveness of IT-administered drugs like baclofen, narcotics, and methotrexate (MTX), though limitations such as restricted brain distribution and potential side effects persist [12,13].

Optimizing NP design to enhance drug stability, targeting specificity and controlled release, is crucial, with animal models aiding in evaluating distribution and therapeutic outcomes. Strategies for CNS drug delivery using engineered NPs focus on BCSFB pathways, especially receptor-mediated transcytosis (RMT), which allows macromolecule transport and is vital for delivering complex therapeutics. Advances in understanding transcytosis mechanisms have created new opportunities for efficient CNS drug delivery.

This review aims to assess the advancements in IT-NP delivery systems designed to circumvent the BCSFB. We will explore the key PK parameters essential for the success of these systems, the mechanisms of NP traversal and persistence within the CNS, and the processes involved in their clearance. Furthermore, we will highlight recent progress in IT-NP delivery strategies, addressing the ongoing challenges and the therapeutic potential of surface modifications and targeted delivery approaches.

By synthesizing the latest research findings, this review seeks to provide valuable insights into the development of advanced IT-NP delivery systems, ultimately contributing to the improved treatment of CNS disorders.

## 2. Materials and Methods

A comprehensive narrative review was conducted by searching PubMed, Google Scholar, and Science Direct for English-language, full-text articles on intrathecal NP penetration through the BCSFB from 1966 to the present. Keywords used included “Intrathecal nanoparticles”, AND “(blood–cerebrospinal fluid barrier)”, AND “(CNS drug delivery)”.

Initially, the search yielded 1307 articles, with 97 selected through database searches. Additional references were manually included based on their relevance, specifically targeting articles not captured by the search engine. These sources comprised recent research articles, clinical trial databases, and relevant industry publications. After screening for title relevance and removal of duplicates, 1210 articles were excluded. The remaining literature underwent manual checks of references for completeness, ensuring the inclusion of pertinent information.

**Figure 1:**
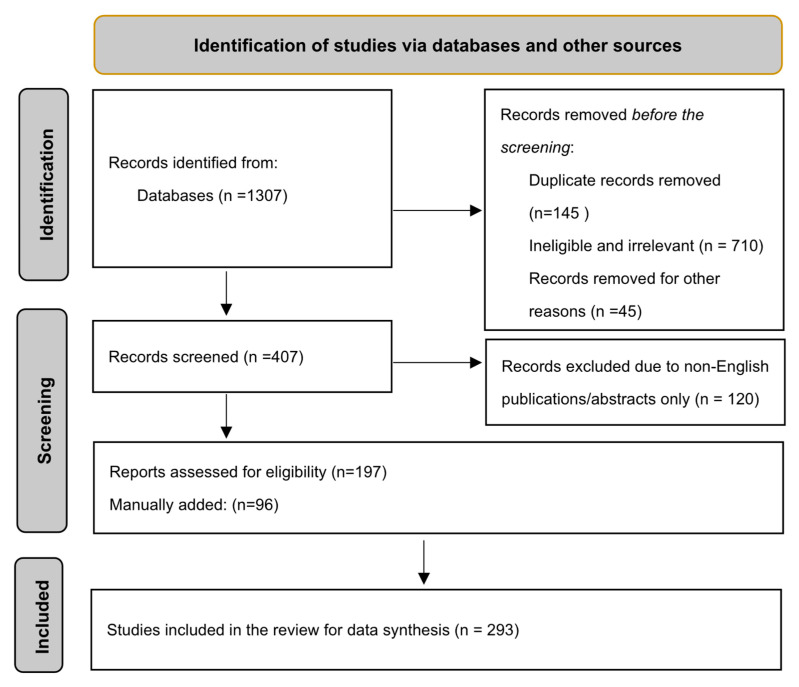
Flow chart of literature search.

Inclusion criteria were English-language, full-text original research articles focused on NP penetration through the BCSFB, and with quantitative data from in vitro and in vivo models assessing BCSFB permeability. Exclusion criteria included non-English publications, conference abstracts, editorials, non-peer-reviewed articles, duplicates, and studies not focused on NP penetration or lacking quantitative data. Preference was given to studies with quantitative data on IT-NP penetration from the CSF into the brain parenchyma, aiming to compile a comprehensive body of literature on NP penetration across the BCSFB for treating neurological diseases. The review synthesizes topics such as the pharmacokinetics of NPs in CSF, the structure and function of the BCSFB, NPs (classifications and properties), penetration of IT-NPs into the brain parenchyma crossing BCSFB, NP uptake pathways, strategies to enhance NP drug delivery across the BCSFB, clinical applications, and future research directions. This approach aimed to compile a comprehensive and representative body of literature on NP penetration for treating neurological diseases and CNS infections. Figure 1 indicates how studies were identified using databases and other sources.

## 3. Results

### 3.1. Pharmacokinetics of Intrathecal NPs in CSF

This narrative review evaluated the effectiveness of the IT administration of NPs, particularly nanomedicines, as an enhanced DDS, in penetrating the brain parenchyma for treating CNS diseases. IT drug administration, which involves the direct infusion of therapeutic agents into the CSF, is gaining recognition as an effective method to bypass systemic barriers to CNS drug delivery. This technique facilitates higher drug concentrations in the CSF surrounding the brain and spinal cord while reducing systemic exposure [14,15]. However, several obstacles, such as poor drug solubility, suboptimal PKs, limited tissue distribution, and potential neurotoxicity, hinder the effectiveness of many IT-administered drugs [16,17]. Consequently, only a few drugs have the necessary biophysical properties for successful delivery via this route. IT administration directly delivers drugs to the CSF, bypassing the BBB to achieve high brain concentrations and minimize off-target exposures and toxicity [18].

Hydrophilic compounds rapidly diffuse through the subarachnoid space (SAS) but are quickly cleared, necessitating frequent dosing to sustain therapeutic levels [19,20]. In contrast, hydrophobic or lipophilic compounds often exhibit poor solubility. Additionally, small molecules tend to partition into lipophilic cell membranes and bind to extracellular matrix proteins, which limits their distribution beyond the injection site and reduces their effectiveness in treating diseases affecting broader CNS regions [21,22,23].

NPs, particularly polymeric and liposomal NPs, are widely utilized to encapsulate therapeutic molecules and address the biophysical and PK limitations of systemic administration [24], and of IT administration as well [25,26]. These versatile NP systems enable the effective encapsulation and prolonged release of various compounds [27]. Engineered for intravenous delivery, NPs can traverse biological barriers through optimized size and surface properties [28].

Intrathecal injection for nanomedicine exhibits unique pharmacokinetics. IT administration disperses NPs within the SAS, facilitating their access to CSF-exposed surfaces of the brain and spinal cord and potentially penetrating the brain parenchyma.

For the distribution of NPs in CSF, the NPs administered intrathecally into the CSF show distinct PK behavior compared to APIs. NPs rapidly disperse throughout the SAS along the entire neuraxis, especially on the ventral surfaces of the brain and spinal cord. This indicates that IT administration is a promising method for the widespread delivery of nanomedicines within the CNS [28]. To deliver drugs to the brain parenchyma, IT administration bypasses the BBB, but the drug is still faced with the other CNS barriers such as the ependymal cells in the choroid plexus (CP) of the BCSFB, which act as barriers, limiting drug action. To overcome the BCSFB and enhance delivery and brain tissue penetration, NPs are being developed and integrated [4]. IT-NPs show promise in treating diseases affecting deep parenchymal targets.

The retention or half-life of NPs within the leptomeninges extends up to 3 weeks or more, unlike the rapid clearance of small hydrophilic molecules [4,28]. This extended retention allows for sustained delivery to the CNS. For example, freely administered cytarabine falls below cytotoxic levels within 24 h of CSF administration [29], whereas the half-life of liposomal cytarabine in the CSF is 43 h [25,26], significantly longer than the 3.4-h half-life of freely administered cytarabine [20]. This extended release profile permits lower peak drug concentrations while maintaining prolonged S-phase targeted cytotoxicity, reducing the required number of injections [30,31]. Nanocarriers extend drug half-lives in the CNS compared to free forms. Cytotoxic agents like methotrexate and cytarabine delivered via multilamellar liposomes sustain therapeutic drug levels in CSF over extended periods [32].

Clearance of NPs from the CSF occurs across the cribriform plate into the nasal mucosa, with a minor portion localizing with nerve roots exiting the spinal column [28]. The glymphatic system and perivascular pathways may also contribute to NP clearance from the brain parenchyma [33]. CSF is produced by the CP and turns over multiple times daily in humans (four times) [34,35]. IT-administered molecules have a half-life of a few hours (h) [20], with clearance posing a major challenge. Studies have shown that NPs can persist in the murine neuraxis at high levels for over three weeks, with most clearance from the brain and minimally from the spinal cord [28]. Clearance of NPs from CNS was observed in a study indicating the NPs’ migration into the nasal sinuses at all examined times [28]. Confocal microscopy revealed no FNP presence in the liver, but they were consistently detected in the spleen, peaking in concentration 2 h post-injection and primarily accumulating around the white pulp [28]. Research on gold nanoclusters indicates that the glymphatic system is the primary pathway for DDS clearance, with exosome transport across the BBB also contributing. Microglia play a vital role in collecting DDS and facilitating their removal via the glymphatic system or BBB [36]. The combined roles of the glymphatic system, BBB, and microglia in DDS brain clearance provide the first evidence of nano-sized DDS elimination from the brain.

### 3.2. Blood–CSF Barrier: Structural Features, Permeability, and Function

The BCSFB, similar to the BBB, is essential for protecting the brain from toxic substances. This barrier is mainly formed by tight junctions (TJs) in the CP epithelium, composed of transmembrane proteins and junctional adhesion molecules, along with cytoplasmic proteins like zonula occludens (ZO) proteins (ZO-1, ZO-2, ZO-3) and non-MAGUK proteins such as cingulin and AF-6 [37,38].

The BCSFB demonstrates greater permeability compared to the BBB due to epithelial pores and vesicles, which create a significant filter for proteins. This allows water-soluble substances, which cannot traverse the BBB, to cross this barrier at rates inversely related to their molecular weight [39]. In a study, the permeability of the BCSFB and the BBB was compared, and it was found that the BCSFB is crucial for CNS homeostasis, regulating the exchange of substances between blood and CSF. The BCSFB is more permeable than the BBB due to pores and vesicles in its epithelial layer, facilitating the movement of specific molecules and water-soluble substances that cannot cross the BBB. While lipid-soluble substances over 500 Da struggle to pass through the BBB, larger hydrophilic molecules can more easily cross the BCSFB [39]. Additionally, ependymal cells in the BCSFB lack TJs, enabling rapid fluid and large molecule exchange, such as proteins [40,41].

CPECs are equipped with various transport proteins and receptors, facilitating the transport of amino acids, hormones, proteins, growth factors, and pharmacologically active agents, and displaying high levels of receptors for molecules such as low-density lipoprotein (LDLR) and serotonin (5-HT) [42,43,44,45]. These cells possess numerous transporters and ion channels, distributed in a polarized fashion, enabling regulated directional movement across the BCSFB [46,47]. Tight junctions at the BCSFB and BBB restrict drug exchange through the paracellular pathway [48].

Table 1 compares the anatomical differences, transporter mechanisms, and pathway distinctions between the BBB and the BCSFB.

The BCSFB comprises choroid plexus epithelial cells (CPECs). Despite having a smaller surface area, CPECs regulate the permeability of nutrients and xenobiotics. The CP is a highly vascularized structure with villi projecting into the cerebral ventricles [51]. Although the CP capillaries are fenestrated, allowing relatively unrestricted movement of water and solutes, a barrier is established by a single layer of polarized epithelial cells linked by tight junctional proteins [52,53]. These tight junctions regulate the passage of molecules and ions, with CPECs primarily responsible for secreting and maintaining the balanced composition of the CSF. In humans, CSF volume averages around 140 mL, with replacement occurring four to five times daily [54]. Additionally, the CSF serves as a drainage system for the brain, diluting and eliminating metabolic byproducts and molecules—a process known as the “sink effect” [55,56]. This effect is particularly pronounced for large-molecular-weight and hydrophilic compounds. Similar to the BBB, CPECs exhibit polarized expression of numerous receptors, ion channels, and transporters [51,57,58,59].

The BCSFB resides in the CP of the brain ventricles, composed of epithelial and endothelial cells (ECs), with highly vascularized stroma featuring fenestrated capillaries surrounded by connective tissue and immune cells [42,60,61]. Cuboidal epithelial cells cover the stromal side facing the ventricles, while phagocytic Epiplexus (Kolmer) cells adhere to this layer, communicating with epithelial cells via pannexin-1 channels [62,63,64]. The CP varies in morphology across brain ventricles, being thin in the lateral ventricles, complex in the fourth, and intermediate in the third [10,65]. Beyond producing CSF and forming the BCSFB, the CP is involved in the circadian regulatory system [65]. The BCSFB has chemosensory receptors for monitoring blood, CSF, and interstitial fluid composition, potentially regulating brain fluid changes [66].

CPECs display polarized expression of receptors, ion channels, and transporters, similar to the BBB [51,57,58,59]. Drug transporters at the BCSFB influence CSF drug concentrations, impacting CNS drug efficacy and toxicity [59,67,68]. However, the tissue-level function of these transporters and their role in brain drug disposition are not fully understood. Unlike CPECs, general ependymal cells lack tight junctions between neighboring cells. Instead, gap junctions and zonulae adherens facilitate the exchange of solutes and macromolecules between the ventricular space and brain tissue [40,41,69,70,71,72,73]. CPECs, specialized ependymal cells, form the CPs suspended in each ventricle. These structures, with highly vascularized stroma and fenestrated vessels, allow fluid and solute exchange [74,75]. Unlike general ependymal cells, CPECs are sealed by tight junctions composed of proteins like zonula occludin-1 and claudins, contributing to the BCSFB [76]. CPECs have well-developed microvilli for enhanced transport processes and smaller cilia compared to ependymal cells, indicating their specialized function in the ventricular system [69,77,78]. Although the CP epithelium and ependyma share a common embryological origin, they are significantly different. The ependyma, composed of cuboidal epithelial cells connected by gap junctions, lines the cerebral ventricles, while the CP epithelium is distinct [79].

The BCSFB, formed by CPECs and tight junctions, regulates CNS homeostasis by selectively allowing the passage of substances between blood and CSF. Its greater permeability compared to the BBB facilitates exchange, impacting drug delivery and neurological health. Understanding these mechanisms is crucial for developing targeted therapies.

### 3.3. Classifications and Characteristics of NPs That Influence Permeability

#### 3.3.1. Classification of NPs

- Lipid-based NPs: Lipid-based NPs encompass liposomes, micelles, solid lipid nanoparticles (SLNPs), and emulsions, valued for their biocompatibility, ability to encapsulate both hydrophilic and hydrophobic drugs, and controlled release capabilities. Among these, liposomes stand out in antibiotic delivery research due to their structural and compositional versatility, which enhances PKs and PDs [80]. Liposomes protect antibiotics, enabling targeted delivery to infection sites while minimizing toxicity to healthy tissues. They interact with bacterial cell walls to increase antibiotic concentration within bacteria, enhancing therapeutic efficacy [80,81].
- Polymeric NPs: Polymeric NPs encompass dendrimers, polymersomes, and polymer micelles. These NPs offer structural flexibility, a high drug-loading capacity, and controlled release profiles. They are particularly useful for delivering drugs that require prolonged and targeted delivery.
- Cell-derived biomimetic NPs: Cell-derived biomimetic NPs include exosomes and stem cell-derived NPs. These NPs mimic natural cellular structures, enhancing their biocompatibility and ability to evade immune detection, making them ideal for delivering therapeutic agents to the CNS.
- Inorganic NPs: Inorganic NPs, such as gold NPs, iron oxide NPs, and mesoporous silica NPs, offer unique properties like magnetic responsiveness and enhanced imaging capabilities. These NPs are used for therapeutic delivery and diagnostic purposes.

#### 3.3.2. Key Properties of NPs Affecting Transport and Distribution

NPs with unique properties like size, morphology, surface charge, hydrophilicity, and modifications affect their distribution and circulation in the body. Prolonging NPs’ half-life is crucial for effective CNS barrier penetration and is a key research focus.

- Size: The size of NPs significantly influences their ability to cross biological barriers. Smaller NPs (<100 nm), especially those around 37–39 nm, are more likely to traverse the BCSFB via transcellular routes such as RMT or adsorptive-mediated transcytosis [82,83]. The effective pore size of the BCSFB is approximately 0.0028 μm, allowing paracellular diffusion of very small NPs and suggesting that 99.8% of the BCSFB’s surface area is involved in transcellular diffusion [84]. NP size is critical for distribution and efficacy. NPs > 20 nm can cross the CNS barriers, while those <5 nm are excreted by the kidneys, and those >200 nm are removed by organs like the liver and spleen [85]. Ideal brain-delivery NPs are <100 nm [83]. For example, 50 nm gold NPs have the highest cellular uptake in HeLa cells [86].
- Surface charge: Surface charge affects NP stability, cellular uptake, and biodistribution [87,88]. Positively charged NPs often exhibit enhanced cellular uptake through electrostatic interactions with negatively charged cell membranes, facilitating their transport across barriers [89]. Zeta potential is a measure of the surface charge of NPs in suspension, influencing their stability and interaction with biological membranes. A high zeta potential (either +/−) typically indicates good stability, reducing the likelihood of aggregation and promoting consistent delivery.
- Surface characteristics: Surface characteristics, like hydrophobicity, hydrophilicity, and targeting ligands, crucially determine NP interactions with biological systems. Surface modifications improve targeting efficiency, reduce off-target effects, and enhance therapeutic outcomes by stabilizing NPs and preventing rapid immune clearance. Rigid copolymer ligands and auxiliary lipids can increase NP robustness [90]. Hydrophobic surfaces improve cell uptake and immune activation [91]. Specific ligands, such as antibodies and peptides, enhance targeting and drug availability [92]. For instance, T7 peptides target the brain by binding to transferrin receptors on glioma cells [93].
- Morphology of NPs: the shape of NPs affects distribution and uptake efficiency. Nonspherical NPs, like nanorods, show better cell entry compared to spherical ones [94]. PEG-modified nanorods are less absorbed by macrophages than nanospheres [95], indicating that shape influences drug delivery and uptake [85,94,95].

Optimizing NPs size, morphology, surface charge, and modifications is crucial for enhancing distribution, targeting, and therapeutic efficacy. Future research should focus on refining these properties to advance NPs-based DDS, as summarized in Table 2 for key BCSFB crossing properties.

### 3.4. Penetration of IT-NPs in CSF across BCSFB

Penetration of NPs from the CSF to the brain parenchyma was traditionally believed to be restricted to the ventricles and SAS, limiting drug distribution to the superficial surfaces of the brain and spinal cord. However, recent insights into CSF dynamics suggest that colloidal carriers and their payloads can penetrate deeper into the brain parenchyma despite some contradictory findings [4].

IT administration using nanocarriers extends drug half-lives compared to free-form drugs, altering their pharmacokinetic profiles due to specific formulation techniques [4]. This method enhances drug concentration in the CSF, potentially improving brain parenchyma penetration. For instance, nimodipine-loaded PLGA microparticles, designed to treat vasospasm post-subarachnoid hemorrhage, achieve significantly higher CSF drug concentrations compared to intravenous delivery, highlighting the benefits of IT administration [4]. Various nanocarriers, including lipid, polymeric, and metallic, are employed to transport small molecules and biomolecules to these critical areas [97]. IT administration effectively distributes colloids throughout the SAS, with lipid vesicles demonstrating rapid neuraxis distribution in rodents, suggesting potential for therapeutic delivery to the brain and spinal cord [98].

Colloidal formulations have garnered significant attention over the past few decades due to their potential to enhance drug delivery. Nanocarriers enable the effective encapsulation and controlled release of therapeutic agents, with surface and size optimization facilitating differential distribution and transport across biological barriers [99]. Ultra-small colloids (sub-10 nm), such as quantum dots and ultra-small G4 dendrimers, have demonstrated the ability to travel from the CSF into the parenchyma in rodents [100]. The surface properties of nanocarriers significantly affect their distribution; for instance, G4 dendrimers entered the parenchyma after IT administration, while G4-C12 remained at the ependymal surface in mice [101]. CSF-administered dendrimers localized with microglia in brain and spinal cord parenchyma in mice and rabbits [102,103]. Citrate-coated iron oxide (VSOP-C184) was delivered to periventricular tissue in mice, but deep targets were not detected due to detection limitations [104]. Also, research since the 1960s has indicated that high-molecular-weight molecules can effectively spread across the brain when delivered via IT lumbar injection. Rieselbach et al. illustrated this phenomenon by showing the extensive distribution of radioactive colloidal gold (Au198) from the lumbar sac into the cerebral subarachnoid and ventricular systems in both human subjects and primates. This distribution primarily occurs due to the bulk flow of CSF [105].

Various inorganic NPs for IT drug delivery have been explored in studies. For instance, Sumner et al. found that intraventricular (IVT)-administered micron-sized iron oxide particles labeled 30% of the neural progenitor cells in rats, suggesting cellular transport [106]. Liu et al. observed significant parenchymal penetration with oligonucleotide-modified iron oxide in mice following IVT delivery [107]. Similarly, 30 nm organically modified silica (ORMOSIL) NPs administered via IVT achieved parenchymal distribution, with confirmed safety and biocompatibility [108].

Liposome NPs also have shown promise in delivering therapeutic agents to deep parenchymal targets. For instance, IT administration of Cannabidiol (CBD) in a nano-emulsion achieved higher brain concentrations and prolonged efficacy compared to oral administration [109]; in this study, two formulations of Cannabidiol (CBD) were administered IT at a dose of 0.05 mg/kg. The CBD nano-emulsion achieved the highest concentration (C_max_) in the brain at 210 ng/g after 120 min (T_max_), while the polymer-coated NPs (PCNPs) reached a C_max_ of 94 ng/g at 30 min. This is significantly higher than the C_max_ of 5.25 ng/g that would be achieved with oral administration at the same dose, demonstrating the effectiveness of IT delivery. The study also found that the use of PCNPs led to faster distribution of CBD from the lumbar segment of the spinal cord to the brain, reaching T_max_ in 30 min compared to 120 min with the nano-emulsion. However, the nano-emulsion resulted in a 3.7-fold higher AUC in the brain over 0–4 h, suggesting that it increased the residence time of CBD in the brain and slowed clearance [109]. Additionally, in rhesus macaque monkeys, liposome-laden lacZ DNA via intracisternal magna (ICM) injection resulted in widespread gene expression throughout the CNS, reaching deep targets in the hippocampus and dentate nucleus [110]. In primates, liposome constituents were detected in deep parenchymal targets, suggesting intact carrier presence [111]. Similarly, widespread parenchymal delivery has also been reported for mRNA-loaded liposomes, protecting their payload from degradation in CSF [112].

Beyond cancer treatments, IT delivery proves effective in neurovascular diseases and neurotrauma. Microparticles encapsulating nimodipine enhance therapeutic efficacy, paving the way for clinical formulations like DepoCyt^®^ [4,23]. Liposomes and cyclodextrin-based systems have shown efficacy in behavioral models but do not address carrier localization, limiting their effectiveness. Behavioral model efficacy has been shown for IT delivery of carrier-encapsulated small molecules, including cyclodextrin-based systems for testosterone, pregnenolone, estrogen, other steroids and fatty acids [113,114], and liposomes for small molecules and genes [115,116]. Several other studies also confirm the effective delivery of NP payloads, like lipids, small molecules, and genes, to deep parenchymal targets, often more extensively than anticipated. For example, liposomes and encapsulated methotrexate, when administered IVT in cynomolgus monkeys, showed more sustained, high concentration, and uniform distribution between ependymal and cortical surfaces compared to freely administered methotrexate [111]. Similarly, IT-administered poly(lysine) dendrimers, dolichol-loaded liposomes, and gadodiamide-loaded phospholipid bicosomes demonstrated rapid neuroaxis distribution following IVT administration [117,118,119]. Moreover, parenchymal delivery of a hydrophobic carbocyanine dye (DiD) depended on lipid composition for solid NPs administered IVT to mice [120]. However, since DiD was not covalently bound to the carrier, it is unclear whether differential distributions resulted from carrier mobility or payload release before tissue entry.

Polymeric NPs are also used for drug delivery to the CNS. Small polycaprolactone (PCL) NPs sized between 37–39 nm exhibit superior distribution within the CNS compared to larger NPs. Mannitol pre-treatment can further improve their uptake [82]. Recent studies have demonstrated effective delivery of fluorescently labeled siRNA and gene silencing in deep parenchymal targets using a polyethyleneimine (PEI) carrier administered IVT to mice [121]. Additionally, an in vivo study reported the use of a linear polyethyleneimine (LPEI)-g-polyethylene glycol (PEG) copolymer-based micellar nanoparticle system to deliver siRNA targeting BACE1 and APP, two key therapeutic targets in Alzheimer’s disease [122]. Fluorescent polystyrene NPs consistently distribute across brain and spinal cord surfaces following IT administration in mice, showing promise for sustained localization within specific anatomical regions [28]. Gene therapy research supports the delivery of nanomedicine payloads to brain tissue. IT administration of PEI complexes, polyplexes, and liposomes has achieved cortical and periventricular transfection, including within the subventricular zone [123]. IT delivery of APIs can also penetrate the brain parenchyma. For example, a study comparing IT and IV administration of the free form of imipenem/cilastatin (IMI/CIL) in rabbits showed that IT delivery consistently resulted in significantly higher concentrations of imipenem in the brain at 0.25, 2, and 8 h post-administration compared to intravenous administration. Specifically, IT IMI/CIL peaked in the brain at 2 h (~4.5 µg/100 mg), maintaining high levels even at 8 h (~4.0 µg/100 mg). In contrast, intravenous IMI/CIL peaked at 2 h but at a level of approximately 30% of IT administration (~1.5 µg/100 mg), declining to nearly undetectable levels by 8 h (~0.2 µg/100 mg) [124]. This demonstrates that IT administration achieves prolonged and higher imipenem concentrations in the brain compared to intravenous administration, which exhibits a rapid decline after the initial peak.

Overall, these findings highlight the potential of IT nanocarrier systems in enhancing drug delivery to deep brain targets, thereby improving therapeutic outcomes for various neurological disorders and CNS infections. While nanoparticles can provide sustained drug delivery, the use of implantable pumps, such as osmotic pumps, offers an enhanced method for long-term IT drug delivery. These pumps, already in clinical use, can reduce the frequency of injections and improve patient compliance. These studies underscore the potential of IT nanomedicine for effective delivery to the parenchyma, presenting new engineering opportunities such as optimizing surface charge, shape, and the attachment of targeting molecules to colloidal carriers. This review indicates that IT nanomedicine holds significant promise for delivering therapeutic agents to deep parenchymal targets via the CSF, leading to prolonged efficacy and enhanced treatment outcomes for various neurological disorders and CNS infections. Table 3 presents the PK parameters of IT-NPs.

### 3.5. Strategies to Enhance NP Penetration and Efficacy across the BCSFB

To improve NP penetration and efficacy across CNS barriers, including the BCSFB, various strategies have been proposed. Exploiting the CPECs’ transport mechanisms could enhance CNS drug delivery via the BCSFB. Despite limited attention and ongoing debates about its suitability, targeting protein/hormone receptors, solute carriers, and amino acid transporters shows promise [125,126].

RMT utilizes antibodies like the anti-transferrin receptor antibody OX26 to enhance the CNS penetration of drugs such as methotrexate, peptides, and tracer proteins [127,128]. Macromolecules conjugated to a peptidomimetic monoclonal antibody targeting the human insulin receptor (HIRMAb) have shown promise, with valanafusp alpha being developed for Mucopolysaccharidosis Type I (MPSI) [129,130,131]. In vitro BCSFB models are valuable for evaluating such antibodies and other RMT targets. Solute carrier-mediated transcytosis (CMT) uses SLC transporters on CPECs to move small peptides and drugs across the cellular barrier [132,133]. Despite some challenges, this method is used by drugs like L-DOPA and gabapentin [125,126]. Nanotherapeutics use NPs to traverse brain barriers via mechanisms such as paracellular pathways, cell-mediated transport, adsorptive-mediated transcytosis, RMT, CMT, and ligand-receptor interactions [134,135,136,137,138]. Various NP platforms, including liposomes, dendrimers, polymeric NPs, and quantum dots, have been developed for CNS delivery [137,138,139,140,141,142]. In vitro BCSFB models assist in understanding NP interactions, assessing biocompatibility, designing selective nanomaterials, and evaluating the safety and efficiency of preclinical formulations, addressing ongoing research needs in neurotherapeutics [143,144]. Other strategies, such as barrier disruptions, have also been employed.

#### Active Targeting Strategies

Active targeting strategies involve modifying nanocarriers with specific molecules such as antibodies or peptides to enhance their ability to cross the barriers efficiently. This approach improves the delivery of therapeutics to the CNS. Notable improvements in CNS specificity and transcytosis efficiency have been observed with these modified systems [145].

- Monoclonal Antibodies: Extensive research has concentrated on antibodies targeting the transferrin receptor to facilitate brain delivery via nanocarriers. This receptor is highly expressed in brain tissues and the microvessel ECs of the BBB [146]. The OX26 antibody, originally developed to target the transferrin receptor, has been shown to enhance the delivery of daunomycin and plasmids through liposomes [147,148,149], as well as peptides via polymersomes in rat models [150]. Similarly, the transferrin antibody 8D3 has demonstrated improved delivery of DNA plasmids in mouse models [151]. Nevertheless, there is a crucial need to develop antibodies targeting the human CNS, as OX26 and 8D3 are specific to rodent transferrin receptors, which limits their translational applicability [145]. The insulin receptor, expressed at the BBB and on glioma cell membranes, along with the epidermal growth factor receptor (EGFR) found in brain tumor cells, are key targets for brain delivery via immuno-liposomes [152,153,154]. The 83-14 antibody targeting the insulin receptor has markedly increased the delivery of liposomes containing antisense oligonucleotides to gliomas [152]. Likewise, immuno-liposomes with the anti-EGFR antibody IMC-C225 have improved the delivery of chemotherapeutic agents to brain tumor cells [153]. However, the 83-14 antibody, initially a mouse anti-human antibody, has shown efficacy only in larger Old-World primates such as Rhesus monkeys, highlighting the necessity of species specificity in targeted delivery systems [154].
- Cell-Penetrating Peptides (CPPs): NPs coated with CPPs offer a potential strategy to enhance CNS barrier selectivity and facilitate drug transport to the CNS. The trans-activator of transcription (TAT) peptide, derived from HIV-1, is particularly notable for this purpose [155]. TAT induces receptor-mediated endocytosis and can be utilized to tag NPs, resulting in increased brain levels of various therapeutic agents such as ciprofloxacin, coumarin, and macromolecules [156,157,158]. Additionally, synthetic peptides have been employed successfully for brain delivery by modifying their sequences to mitigate inherent biological activities that may cause adverse effects [159,160,161,162]. Recent advancements include the use of novel peptide-based carriers known as Angiopeps for brain drug delivery [163]. These peptides, derived from the Kunitz domain, have demonstrated higher transcytosis rates and parenchymal accumulation compared to other targeting moieties like avidin and lactoferrin. While the exact mechanism of Angiopeps’ cell penetration remains to be elucidated, it is likely mediated by the LDL receptor-related protein-1 (LRP1).
- Targeting with Endogenous Molecules: Apolipoproteins, such as apolipoprotein A (ApoA) and apolipoprotein E (ApoE), have been effectively utilized to target LDL receptors at the BBB. Non-ionic surfactants, particularly polysorbates, promote ApoE adsorption on nanocarrier surfaces, enhancing their targeting capabilities [164,165]. Alternatively, nanocarriers can be directly conjugated to apolipoproteins. For instance, Michaelis et al. demonstrated that direct conjugation of human serum albumin NPs to ApoE via covalent linkages resulted in superior therapeutic effects and prolonged efficacy compared to indirect approaches using albumin with ApoE adsorbed on the surface [166]. Various other substrates, including thiamine, transferrin, folate, glycosides, and lactoferrin, have been evaluated for targeting receptors at the BBB [167,168,169,170,171,172,173]. Although generally less specific than monoclonal antibodies, these substrates offer the advantage of being endogenous molecules present in the human body, potentially reducing the risk of severe immunogenic responses or adverse effects.

Enhancing NP penetration and efficacy across the BCSFB involves various strategies. These include exploiting transport mechanisms such as RMT and solute carrier-mediated transcytosis, employing nanotherapeutics, inducing barrier disruption, and active targeting with endogenous molecules. These approaches show significant promise for improving CNS drug delivery. Alternative strategies for CNS nanomedicine include the following: efflux transporter inhibitors, nanocarrier cationization, paracellular transport enhancement, intranasal administration for olfactory transport of NPs, and the use of focused ultrasound with microbubbles in conjunction with NPs, etc. These innovative methods collectively aim to overcome CNS barriers, offering new avenues for effective drug delivery to the brain.

### 3.6. Clinical Application of IT-NPs in CNS Diseases

Currently, effective therapies for many CNS diseases are lacking, primarily due to the poor accessibility of drugs to the CNS. It is estimated that systemic delivery is ineffective for over 98% of small molecules and nearly 100% of large molecules [7]. Consequently, several CNS diseases remain untreatable.

Mechanisms to improve NP penetration and efficacy across CNS barriers, such as RMT and CMT, have shown promise. Research from four decades ago revealed that large molecules could enter the brain parenchyma from the CSF [174]. However, it is only in recent studies that the biologically significant portion of therapeutic macromolecules or particles, such as gene vectors, delivered to the CNS through the CSF, has been recognized [175].

In clinical settings, a NPs-based DDS offers promising avenues for overcoming CNS barriers to delivering therapeutics to the CNS. Existing clinical applications of NPs via systemic administration, such as Abilify Maintena^®^ (Otsuka) (Aripiprazole—Schizophrenia) [176], Invega Trinza^®^ (Janssen) (Paliperidone palmitate—Schizophrenia), Aristada^®^ (Alkermes) (Aripiprazole lauroxil—Schizophrenia), Sublocade^®^ (Indivior) (Buprenorphine—Opioid use disorder), and Invega Sustenna^®^ (Janssen) (Paliperidone palmitate—Schizophrenia) highlight their potential [134,135,136]. NPs can traverse intact or impaired brain barriers through various mechanisms, including paracellular transport, cell-mediated transport, adsorptive-mediated transcytosis, RMT, CMT, and ligand–receptor interactions. The design of nanocarriers, such as liposomes, dendrimers, polymeric NPs, micelles, and others, allows them to efficiently navigate these pathways [137,138].

IT-NP delivery is a promising strategy for treating CNS disease including CNS infections and other disorders, bypassing the BBB and BCSFB, which restrict the passage of many drugs. IT administration, the direct injection of substances into the CSF, can achieve high concentrations at target sites while minimizing systemic exposure [10]. However, optimizing the distribution of these drugs within the CSF remains challenging [18,21]. The encapsulation of therapeutic molecules within polymeric or liposomal NPs can overcome the biophysical and PK limitations of systemically administered agents [24,177], but IT administration of free drugs faces significant challenges. Hydrophilic drugs are rapidly cleared due to CSF turnover, while hydrophobic drugs tend to remain localized near the injection site. Additionally, macromolecules have difficulty penetrating brain tissue (parenchyma) [178]. Colloidal carriers composed of lipids and polymers have demonstrated potential in delivering genes, imaging agents, and therapeutic molecules throughout the CNS [179]. These carriers not only enhance distribution but also facilitate delivery to deeper brain regions, providing significant advantages over traditional IT therapy.

The concept of IT-NP delivery dates back to 1978 when Kimelberg et al. demonstrated enhanced CNS penetration of liposome-encapsulated methotrexate in primates and reduced peripheral clearance of liposome-encapsulated methotrexate compared to free methotrexate in cynomolgus monkeys following IVT injection [111]. Since then, numerous IT-NPs have progressed to clinical trials and use. For instance: DepoCyt^®^: Liposomal cytarabine, FDA-approved for lymphomatous meningitis, maintaining cytotoxic CSF levels for up to 14 days [25,26,29,30,180,181]. HP-ß-CD: Used for Niemann-Pick Type C disease, stabilizing neurologic symptoms but potentially causing ototoxicity [182,183,184,185,186,187,188], EXPAREL^®^: Liposomal bupivacaine for postoperative pain management, offering extended-release profiles and reducing opioid requirements [189,190,191,192,193], and EG-1962: PLGA-encapsulated nimodipine for aneurysmal subarachnoid hemorrhage, although recent trials were terminated due to unmet endpoints [194,195,196,197].

Ongoing research aims to optimize NP properties such as size, surface characteristics, charge, and lipophilicity to enhance BCSFB crossing and CNS drug delivery. Further understanding of CSF and interstitial fluid dynamics will aid in manipulating NPs distribution within the CNS, enhancing therapeutic outcomes across various CNS pathologies. This growing body of knowledge supports the continued development of NP-based therapeutics for clinical application [198]. The list of clinically used IT-NPs is in Table 4.

## 4. Discussion

This review emphasizes the significant advancements in IT-NP delivery systems aimed at bypassing the BCSFB. These systems have been shown to enhance drug penetration and retention within the CNS, addressing the challenges posed by the BBB and BCSFB. Key findings include the ability of engineered NPs to extend drug circulation times [4], improve CNS penetration, and facilitate sustained release, which is critical for achieving therapeutic efficacy in treating CNS disorders. Recent advancements in nanotechnology have led to the development of diverse nanocarriers sized between 1 and 100 nm, including polymeric nanoparticles (PNPs), SLNPs, liposomes, and micelles, tailored for treating neurological disorders [199,200,201,202,203]. More sophisticated nanosystems such as dendrimers, nanoemulsions, nanogels, nanosuspensions, and nanotubes exhibit superior potential compared to earlier delivery systems. Given the average size of human cells (10–20 µm) and the minimal diameter of blood capillaries (6–9 µm), nanomaterials benefit from their nanoscale dimensions, facilitating efficient transport and internalization by brain capillary ECs via endocytosis and transcytosis [204].

### 4.1. Selection and Challenges in NPs for DDS

The selection of suitable nanocarriers for DDS is critical to the effective delivery of therapeutic agents to the CNS. NPs offer a range of advantages over traditional drug delivery methods, including enhanced drug penetration, extended circulation times, and targeted delivery capabilities. However, the development and application of NPs for DDSs face numerous challenges that must be addressed to optimize their efficacy and safety. The effective delivery of CNS drugs necessitates the selection of nanocarriers with optimized size, surface area, charge, and morphology, alongside properties such as biodegradability, non-toxicity, biocompatibility, cost-effectiveness, and site-specificity [205]. Various nano-drug systems including polymers, micelles, liposomes, dendrimers, nanocrystals, and SLNPs have been employed to enhance drug efficacy, safety, PKs, and PDs [206,207]. Key objectives in nanomedicine development include ensuring safety, achieving high efficacy through targeted drug delivery to minimize off-target toxicity, and improving PKs through sustained drug release. Determining the most promising and safe nanoparticulate DDS among options like polymers, micelles, liposomes, dendrimers, nanocrystals, and SLNPs is challenging. Factors such as size, shape, composition, surface charge, monomer molar ratio, drug solubility, physiochemical properties, and release in cellular environments significantly influence their PKs [208]. Targeted drug delivery aims to reduce side effects, with brain-targeted delivery achievable through receptor-mediated, transporter-mediated, and pharmacological disruption of the BBB. Strategies include decorating delivery systems with BBB receptor ligands, coupling antibodies, peptides, or aptamers with BBB receptor ligands, and using transporter ligands coupled with micelle delivery [209].

NPs have demonstrated several PK advantages over traditional drug delivery methods. As mentioned earlier, engineered NPs can remain in the leptomeninges for extended periods, often exceeding three weeks, which allows for sustained delivery to the CNS [4]. Studies have shown that NPs can distribute rapidly throughout the SAS and penetrate CSF-exposed surfaces of the brain and spinal cord. For instance, small-size PCL-NPs significantly enhanced CNS distribution, with smaller NPs achieving better uptake and widespread distribution within the brain. The mechanisms of NP traversal across the BCSFB involve several pathways, including passive diffusion, carrier-mediated influx, and transcytosis. Transcytosis, in particular, has been identified as a crucial mechanism for NP delivery [210]. Recent research indicates that tuning the avidity of NPs to targeted receptors can optimize RMT. For example, NPs decorated with transferrin showed that proper optimization of transferrin levels is essential for efficient BBB transcytosis and subsequent brain tissue penetration. Despite these advancements, IT-NP delivery systems face several challenges and limitations. These include poor drug solubility, limited tissue distribution, potential neurotoxicity, and rapid clearance from the CSF [10]. Additionally, the extent of NP penetration into deeper brain parenchyma remains uncertain and highly dependent on the NPs’ properties, such as size, surface charge, and shape. Ensuring consistent and controlled release while minimizing toxicity also presents significant hurdles.

While the selection of suitable NPs for DDS presents numerous challenges, ongoing research and technological advancements hold promise for improving CNS drug delivery. By addressing the limitations and optimizing NP properties, more effective and safer treatments for CNS disorders can be developed. Understanding the mechanisms by which NPs traverse the BCSFB is crucial for enhancing their delivery to the CNS. The BCSFB, formed by the choroid plexus epithelial cells, serves as a selective barrier, regulating the exchange of substances between the blood and the CSF. This barrier, along with the BBB, poses significant challenges to the delivery of therapeutic agents to the CNS. Therefore, elucidating the pathways through which NPs can cross the BCSFB is essential for the development of effective DDS targeting CNS disorders.

### 4.2. Toxicity of Nanoparticles

The increasing use of NPs in medical applications, particularly for drug delivery to the CNS, necessitates a thorough understanding of their potential toxicity. While NPs offer significant advantages in targeting and treating CNS disorders, their small size and unique properties can pose risks to human health [211]. Several factors influence the toxicity of NPs, including their size, shape, surface charge, and chemical composition [211].

Scientists have proposed that NPs smaller than 10 nm can easily penetrate human tissues and disrupt normal cellular biochemical environments [212]. Studies in animals and humans have shown that, after inhalation or oral exposure, NPs distribute to the liver, heart, spleen, brain, lungs, and gastrointestinal tract [213]. The immune system is activated to clear these NPs, with an estimated half-life of about 700 days in human lungs, posing a persistent threat to the respiratory system. During metabolism, some NPs congregate in liver tissues [214,215]. NPs are more toxic than larger particles of the same substance, with toxicity generally being inversely proportional to their size [216]. NPs can lead to unpredictable health outcomes due to their unique properties. Bridging the knowledge gap on NP toxicity is crucial for safe nanotechnology applications. Future research should standardize toxicity assessments, investigate long-term effects, and explore impacts on various ecosystems and organisms.

### 4.3. NP Uptake Pathways across the BCSFB

NPs enter cells through either passive or active methods. Passive diffusion, which includes transmembrane and paracellular transport, is restricted to small, uncharged molecules and is less effective for drug delivery. Therefore, NPs are predominantly absorbed by cells via active transport mechanisms such as carrier-mediated, absorptive-mediated, and RMT [217]. Among the transcytosis pathways, RMT is more predominant in the transport of NPs across the BCSFB as described below.

Transcellular transport across barriers primarily occurs via transcytosis, especially for large molecules crucial for brain function such as polypeptidic hormones, metal carriers, and lipoproteins. Transcytosis involves sequential steps including endocytosis, where extracellular cargo is internalized into vesicles from the cell membrane, followed by sorting, trafficking through the endosomal network, fusion with the target membrane, and release into the extracellular space [218,219,220,221]. Pericytes regulate BBB permeability by directing endosomes for degradation, reducing non-specific transcytosis [222,223]. In contrast, the BCSFB shows high endocytic activity [224,225,226]. This suggests its potential for transcytosis of therapeutic agents. However, the exact role of this activity in transporting molecules across the BCSFB remains unclear [227,228].

Endocytosis, the initial step in transcytosis, is an energy-dependent process mediated by various mechanisms. Clathrin-coated pits and caveolin-1-containing lipid rafts are two primary sites for vesicle formation on the plasma membrane, facilitating RMT. Clathrin-coated pits are enriched in cell surface receptors that trigger endocytic events upon ligand binding, while caveolae are involved in receptor-mediated, fluid-phase, and adsorptive endocytosis. Additionally, vesicles can form from non-clathrin and non-caveolin lipid rafts, contributing to endocytotic activity [218,219,220,221]. Early electron microscopy studies suggested low vesicular density in cerebral endothelium compared to other endothelia, with limited transcytosis of fluid-phase endocytosis tracers under non-pathological conditions [221]. Recently, pericytes have been identified as key regulators of BBB permeability, downregulating a non-specific transcytotic mechanism, possibly initiated by fluid-phase endocytosis [222]. In contrast, choroidal epithelial cells exhibit a high density of vesicles, indicative of robust endocytotic activity at the BCSFB [224,225].

Functional annotation of highly expressed gene sub-datasets in human and mouse choroidal epithelium cells identified significant canonical pathways associated with endocytosis [226]. This heightened endocytotic activity supports the metabolic and synthetic functions of the BCSFB, although its relevance to the transcellular transport of macromolecules remains unclear [227,228]. Transcytosis pathways are of considerable interest in CNS drug delivery, offering the potential for delivering large cargoes such as biotherapeutic agents, nanobodies, liposomes, and NPs [210]. Cationization approaches have been developed to promote adsorptive-mediated endocytosis of therapeutic agents, particularly using cationic CPPs [229]. However, their lack of selectivity limits their potential for brain delivery [229]. RMT offers organ selectivity by targeting receptors highly expressed in barrier cells [230,231,232]. Various vectors, including antibodies and noncompetitive peptide ligands, have been explored to enhance RMT, although careful optimization is required [233]. Understanding transcytosis mechanisms is crucial for targeted CNS drug delivery. Despite challenges, these pathways hold promise for delivering important molecules to the brain, with RMT being particularly effective for NP transport across the BCSFB.

#### 4.3.1. Receptor-Mediated Transcytosis

NPs functionalized with specific ligands can bind to receptors on the endothelial cell surface, triggering internalization and transport across the barrier. RMT includes the transferrin receptor pathway, the insulin receptor pathway, and the low-density lipoprotein receptor pathway. LDL receptor-related protein family pathways are common mechanisms utilized by both the BBB and BCSFB. In contrast, the folate pathway, responsible for folate transport across the choroidal epithelium, and plasma protein transport are specific to the BCSFB. In this method, special receptors on the cells of the BBB and BCSFB facilitate the transport of large molecules such as transferrin or insulin into the brain. This process, known as RMT, involves the binding of the ligand to the receptor on the blood side of the brain barrier. Subsequently, the ligand is internalized, transported across the barrier, and released into the brain parenchyma or the CSF [234].

- Transferrin Receptor Pathway: The transferrin receptor TfR1 has been a primary target for enhancing the delivery of compounds to the brain due to its selective expression in the cerebral microvessel endothelium relative to other endothelia. TfR1 is also present in the BCSFB and has been identified in both rat and human CP epithelia [235,236,237]. The mechanism of TfR1-mediated iron delivery into cells is well understood. Iron-loaded transferrin binds to TfR1 at the clathrin-coated pits on the cell membrane, and the complex is internalized by endocytosis. Iron is released from transferrin in acidifying endosomal vesicles and is exported to the cytosol for metabolic functions or storage. TfR1 is recycled to the cell membrane, releasing iron-free transferrin with low affinity at neutral pH. This route of iron delivery to the brain requires the export of cytosolic iron at the brain-facing membranes of BBB and BCSFB cells, possibly involving ferroportin or another mechanism [238]. Recent studies suggest that TfR1-mediated transcytosis may occur in the choroidal ependymocytes, although the exact mechanism remains unclear. Evidence supporting this comes from experiments utilizing a novel engineered receptor/ligand system expressed specifically in this barrier [239]. The mechanisms underlying transcytosis, particularly the triggers for endocytosis and vesicular pathways, remain poorly understood. Moreover, the impact of TfR1-mediated transcytosis at the BCSFB on antibody-based therapeutic drug delivery targeting the BBB is still being investigated. Furthermore, the canonical endocytosis pathway involving TfR1 recycling could be utilized in strategies for CNS delivery, as demonstrated with gold NPs [240]. Transferrin-conjugated NPs with a pH-sensitive linker showed enhanced brain penetration in mice post-systemic administration compared to non-cleavable linker-bound NPs. Acidic endosomal pH likely dissociates gold particles from stable transferrin-TfR1 complexes, aiding brain access. Further research is needed on particle sorting and release mechanisms at the endothelial abluminal membrane. This effective delivery method in the choroidal epithelium warrants exploration at the BCSFB.
- The Insulin Receptor Pathway: Insulin transportation through the BBB relies on RMT [241]. Inspired by the OX-26 anti-TfR1 antibody model, a monoclonal antibody was developed against the human insulin receptor. This antibody demonstrated endocytosis in human cerebral capillaries and swift transcytosis in non-human primate brain parenchyma [242]. Consequently, an antibody-based delivery platform emerged, facilitating the engineering of recombinant bifunctional fusion proteins to transport therapeutic proteins, like growth factors or enzymes, across the BBB [243]. Fusion constructs coupling a humanized anti-insulin receptor monoclonal antibody with lysosomal enzymes are currently in phase I clinical trials for lysosomal storage disorders affecting the brain (according to the NCT02262338 Health USNIO, ClinicalTrials.gov registry and results database 2016). Previous investigations into the distribution of insulin receptors in the brain revealed a high insulin-binding capacity not only at the BBB but also in the CP [244,245]. The CP was identified to possess the highest density of insulin-binding sites among all brain structures [245,246]. Further confirmation of insulin receptor gene expression in the CP was obtained through in situ hybridization [247]. Although direct evidence of insulin RMT across the BCSFB is lacking, continuous blood infusion of insulin in dogs and humans raised the CSF level of the hormone concurrently with the plasma level [248,249]. Modeling of insulin uptake kinetics in CSF from plasma suggested the existence of an intermediate compartment between blood and CSF, possibly corresponding to parenchymal interstitial fluid or CP tissue [248,250]. The rapid elimination of insulin from the CSF following IVT perfusion, relative to the elimination of a CSF bulk flow marker, indicated that insulin receptors in the BCSFB could mediate the CSF-to-blood transcytosis of the hormone, contributing to insulin signal termination [251]. In summary, the exploration of insulin RMT offers promising avenues for the development of antibody-based delivery platforms, potentially revolutionizing therapeutic interventions for brain disorders.
- The LDL Receptor Pathway (A Gateway for Cholesterol Delivery to the Brain): This passage highlights the low-density lipoprotein (LDL) receptor’s role in brain cholesterol homeostasis. The LDL receptor, a high-affinity cell surface protein, binds LDL particles (carrying cholesterol) via apolipoprotein B. It then facilitates their internalization through coated pits. These LDL particles are delivered to lysosomes for degradation, releasing cholesterol for cellular use. Importantly, the LDL receptor is more abundant at the BBB compared to other ECs. This strategic positioning allows it to mediate the transcytosis of LDL particles, delivering cholesterol to brain cells [252]. Research has identified specific peptide ligands that bind the human LDL receptor’s extracellular domain using phage display biopanning [233]. These optimized peptides exhibit high affinity for the receptor without competing with endogenous LDL. In vivo, studies using biphoton microscopy demonstrated that these peptides can extravasate from blood vessels in the spinal cord and accumulate in the surrounding brain tissue. Conversely, a scrambled control peptide remained confined within the blood vessel lumen. Further investigation is necessary to understand the transcytosis mechanism better and evaluate its potential for delivering therapeutic cargo to the brain. Limited information exists regarding LDL receptor expression at the BCSFB. While transcripts have been detected in the mouse CP (Allen Institute for Brain Science), recent immunohistochemical analysis of human CP tissues revealed consistent expression of the receptor in choroidal epithelial cells across all seven patients tested [253]. Future studies exploring LDL-RMT for CNS drug delivery should consider investigating both the BBB and BCSFB in parallel.
- The LDL Receptor-Related Protein Family: LDL receptor-related proteins (LRPs) are a family of cell surface receptors involved in endocytosis and transcytosis of macromolecules across barrier-forming cells [254]. They have been explored as potential targets for drug delivery to the CNS [255,256]
  (A) LRP1 and the BBB: LRP1 was initially considered a promising target for brain drug delivery due to its ability to bind Kunitz protease inhibitor domain-containing peptides like angiopep2 [256]. However, LRP1 expression in human BBB endothelium is debated. While some studies detected LRP1 in mouse brain microvessels, others failed to find it in human brain tissues [257,258,259].
  (B) LRP1 and the BCSFB: LRP1 is consistently expressed in the CP, a structure responsible for producing CSF [253]. This suggests a role for LRP1 at the BCSFB. Several LRP1 ligands are present in CSF, and LRP1 may be involved in their clearance and protease activity homeostasis [260,261,262].
  (C) LRP2 and LRP8 as Potential CNS Drug Delivery Targets: LRP2 and LRP8 are other LRP family members with potential for CNS drug delivery. LRP2 is highly expressed in the CP throughout life and mediates transcytosis of leptin and insulin-like growth factor I from blood to CSF [224,263]. LRP8 is also highly expressed in the CP and shows apical localization, suggesting a role in CSF transport [264,265]. LRP8 knockout mice have lower brain selenium levels, suggesting its involvement in brain selenium uptake [266]. While LRP1’s role at the BBB remains unclear, LRP1, LRP2, and LRP8 in the CP highlight their potential for targeted CNS drug delivery. Further research is needed to understand LRP-mediated transcytosis mechanisms and develop specific peptide ligands or other triggers for an LRP-based DDS. The LRP receptors in the BBB and BCSFB are summarized in Table 5 below:
- The Folate Pathway: Folates, essential vitamins for vital metabolic processes, require facilitated transport across cell membranes due to their poor permeability at physiological pH. Three distinct systems have been identified for this purpose, each characterized by varying affinities for the physiologically active form of folate, 5-methyltetrahydrofolate (5MTHF), and differing pH preferences. Notably, two of these systems are classified as facilitative transporters, belonging to the extensive solute carrier superfamily [49]. The reduced folate carrier (RFC, SLC19A1) is a broadly expressed transporter with low affinity, operating effectively at normal pH levels and mainly found in the choroidal epithelium’s apical membrane. Conversely, the proton-coupled folate transporter (PCFT, SLC46A1) functions optimally at acidic pH, with lower affinity, primarily in the intestinal epithelium but also in the CP. PCFT immunolabeling reveals staining in both basolateral membranes and cytoplasm [267,268]. Folate receptors (FR) facilitate folate endocytosis at neutral pH without clathrin, with FR-alpha (FRα) mainly in specialized epithelia, notably the choroidal epithelium, showing intense immunoreactivity, especially in the human CP [267,268]. FRα exhibits a low binding constant in the nanomolar range, akin to plasma concentrations. CSF folate levels are 3- to 4-fold higher than blood. FOLR1 gene mutations, causing cerebral folate deficiency, lower CSF folate but not blood levels. FRα, present only in the CP, is crucial for folate delivery. The proposed pathway posits the following under normal conditions: (1) FRα-mediated endocytosis facilitates basolateral membrane folate uptake, (2) within acidifying endosomes, folates are released from FRα and exported by PCFT, and (3) RCF transports folates across the apical membrane into CSF [269]. Recent research employing both in vitro and in vivo methods unveiled a new folate delivery mechanism across the choroidal epithelium [267]. In Z310 rat choroidal cells expressing human FRα (hFRα), fluorescent folates and FRα were co-transported from the basolateral to the apical membrane and released into exosomal vesicles. This exosome-mediated delivery was supported by hFRα presence within intraluminal vesicles of multivesicular bodies. IVT injection of hFRα-positive exosomes from transfected Z310 cells into mice resulted in their penetration into the brain parenchyma, particularly astrocytes, away from the ventricular wall, while hFRα-negative exosomes remained at the periventricular border. FRα-containing exosomes were also found in human CSF, correlating with CSF levels of 5MTHF. The CP significantly contributed to CSF exosomes, with 38% being FRα-positive in control individuals. This novel mechanism, coupling RMT with targeted distribution via exosomes, holds promise for cerebral drug delivery. Additionally, cancer cells, notably pediatric ependymal tumors, frequently overexpress FRα [270]. Folate-conjugated anticancer agents entering the CSF via the CP can directly target tumors, enhancing penetration into the target cells.
- Plasma Protein Transport: Decades ago, plasma protein transfer from blood to CSF in newborn rats, demonstrated using labeled albumin and immunoglobulins, was attributed to the immaturity of the BCSFB [50]. However, the current understanding largely disregards this explanation, with accumulating evidence supporting a specific and developmentally regulated mechanism of protein transfer across the choroidal epithelium. Immunoreactivity of various plasma proteins was observed in choroidal epithelial cells during fetal life in multiple mammalian species, including humans [271,272,273]. Actual protein transfer from blood to CSF was confirmed using exogenous human albumin in sheep fetuses or rat neonates [274,275]. This protein uptake and transfer pathway by the CP appears to be specific, as not all plasma proteins can be detected within choroidal epithelial cells [274]. Moreover, the lack of correlation between CSF/plasma concentration ratios and the molecular radius of transported proteins suggests the involvement of a receptor-mediated transfer mechanism [273,276]. Extensive research has aimed to identify choroidal receptors for plasma proteins and characterize the cellular mechanism supporting transport to the CSF. Given the structural dissimilarity of transported proteins, multiple pathways are anticipated [277]. For instance, bovine fetuin administered to Monodelphis fetuses was taken up by choroidal epithelial cells following intraperitoneal injection but not after IVT administration, suggesting a unidirectional transfer mechanism from blood to CSF, unless influenced by physiological concentration gradients [271]. The BCSFB transporters and receptors relevant to drug delivery are summarized in Table 6.

#### 4.3.2. Carrier-Mediated Transcytosis

Transporters in the microvasculature of the CNS barriers are crucial for drug transport by specifically recognizing and binding to drugs. Glucose transporters (GLUTs) are key soluble carriers [41] and are highly expressed in mammalian ECs [280]. In pathological conditions such as Alzheimer’s disease, GLUT1 levels decrease in cerebral capillaries, reducing glucose uptake in the brain and leading to cognitive decline. GLUTs may serve as effective carriers for neurotherapeutic drugs aimed at treating neurological and neurodegenerative diseases. Studies have explored the use of ligand-conjugated nanocarriers, including multivalent glucoside-coupled liposomes [281], mannose-derived liposomes [282], glucose-coated gold NPs [283], and 2-deoxy-d-glucose functionalized NPs [284]. These nanocarriers specifically bind to GLUTs, enhancing drug penetration across the BBB and increasing drug levels in the brain.

#### 4.3.3. Absorptive-Mediated Transcytosis

Positively charged molecules and negatively charged endothelial cell cytoplasmic membranes can overcome the barrier of the BBB through electrostatic interactions, facilitating the specific transport of drug molecules to the brain. Macromolecular drugs and NPs conjugated with cationic ligands can penetrate brain parenchymal tissues effectively. Absorptive-mediated transcytosis (AMT) involves cationizing proteins by amidating their carboxyl groups with natural or synthetic diamines and polyamines [285]. This technique has been applied to various proteins such as albumin [286], anti-amyloid peptide antibodies [287], and nerve growth factor [287] for diagnostic and therapeutic purposes.

Furthermore, CPPs are strategically used to transport hydrophilic neuropharmaceuticals across ECs via nonreceptor-mediated endocytosis. CPPs, which are cationic peptides, bind to the anionic cell membranes of microvascular endothelia to deliver drugs and genes to specific brain sites [288]. Over the past decade, CPPs have proven effective in delivering potential therapeutic drugs to the brain. For instance, SynB, a peptide derived from a natural protein, significantly increased the brain uptake of doxorubicin (DOX) by inhibiting P-glycoprotein (P-gp)-mediated efflux, compared to unmodified DOX [289].

There are some contradictory findings and debates; for example, some of the literature says that despite bypassing the BBB and the CSF barrier, the brain parenchyma remains separated from the CSF by a layer of ependymal cells and the glia limitans. This brain–CSF barrier has a significantly smaller surface area compared to the capillaries of the CNS, thus restricting the diffusion of drugs from the CSF into the brain parenchyma [290]. Moreover, the rapid turnover of CSF further diminishes the effectiveness of IT or IVT drug delivery for treating parenchymal diseases. However, there is ongoing debate regarding this issue, with some articles suggesting that IT injection may indeed bypass the BBB and the BCSFB. The clinical implications of these findings are profound. Enhanced NP delivery systems hold promise for treating a range of CNS disorders by improving drug concentration and distribution within the CNS, thus potentially enhancing therapeutic outcomes. For example, IT administration of CBD nano-emulsions achieved significantly higher brain concentrations compared to oral administration, demonstrating the potential for IT-NPs to achieve superior PK profiles [109]. Using an implantable osmotic pump can effectively replace frequent IT injections or lumbar punctures by offering sustained drug release over extended durations [291].

Future research should optimize NP properties for enhanced CNS targeting and minimal adverse effects, alongside evaluating the long-term safety and efficacy of IT-NP delivery in clinical settings. Surface modifications and targeted delivery strategies should be explored to enhance NPs’ effectiveness and specificity. Understanding NPs’ clearance mechanisms via the glymphatic system and perivascular pathways is crucial for developing efficient IT-NP systems. In summary, IT-NP delivery holds promise for overcoming BBB and BCSFB barriers and improving CNS drug delivery. Ongoing research to optimize NP properties and delivery mechanisms is key to enhancing treatments for CNS disorders and advancing this field.

## 5. Conclusions

This review underscores the potential of IT-NP delivery systems for treating CNS disorders and infections, highlighting the ability of various NPs to penetrate the brain parenchyma across the BCSFB. The literature indicates that NPs’ brain penetration is influenced by their properties and functionalization, with the BCSFB being more permeable than the BBB. Optimal NP properties for crossing the BCSFB include a size range of 37 to 39 nm and a positive charge.

IT-NP delivery achieves higher and prolonged drug concentrations in the brain, improving distribution and minimizing systemic exposure. Various NPs, including lipid-based, cell-derived biomimetic, inorganic, and polymeric types, show promise in overcoming the BCSFB. Liposomal NPs are particularly effective for treating infections.

Despite these advances, challenges in safety, efficacy, and regulatory approval remain. Future research should address these development challenges to bridge the gap between academic research and industrial acceptance.

## Figures and Tables

**Table 1:** Differences between the BBB and BCSFB.

Aspects	BBB	BCSFB	References
Anatomy	1- Layer of EC interconnected by TJs 2- Basal membrane (consists of basal lamina of astrocytes and basal lamina of EC) 3- Pericytes 4- Astroglial endfeet	1- Choroidal epithelial cells interconnected by gap junctions 2- Basal membrane 3- Endothelium of the pia mater capillaries containing fenestrations	[39]
	5- Ependymal Cell have TJs	Ependymal Cell do not have TJs	[40,41]
Transporter and Pathways	1- No fenestrations 2- Passive diffusion (small molecules and lipophilic substances) 3- Facilitative and energy-dependent transport (e.g., glucose and amino acids) 4- Ion channels and exchangers 5- Reduced pinocytosis 6- Entrance of immune cells possible 7- TfR pathway, Insulin receptor pathway, Lipoprotein receptor pathway, Glucose and LRP	1- Several fenestrations 2- Diffusion of plasma solutes 3- Active receptor-mediated transport 4- Ion channels and exchangers (e.g., Na/K ATPase located in microvilli of the CP) 5- Pinocytosis 6- No entrance of immune cells (discussed but the evidence is still missing) 7- TfR pathway, Insulin receptor pathway, Lipoprotein receptor pathway, Glucose receptor pathway, LRP, Folate receptor pathway, and Plasma protein receptor pathway.	[39,43,44,49,50]

Abbreviations: Tight junctions: TJs, Transferrin receptor: TfR, Endothelial cells: EC, Lipoprotein receptor-related protein: LRP.

**Table 2:** Key properties of NPs essential for crossing the BCSFB.

Type of NPs	Size of NPs	Charge and Lipophilicity	Surface Properties	Shape	References
PEGylated PCL-NPs	37~39 nm	Negative	Not functionalized	NA	[82]
Au NPs	50 nm	NA	NA	NA	[86]
INS-GNPs	20 nm	NA	NA	NA	[83,85]
Nanorods (rod-shaped)	NA	NA	NA	Non-spherical	[94]
Gold NPs	NA	Positively charged	NA	NA	[96]
PEG-modified nanorods	NA	NA	NA	Nanorods	[95]
INS-GNPs	100 nm	NA	NA	NA	[83]

Abbreviations: Not available: NA, Polycaprolactone: PCL, Insulin-coated gold nanoparticles: INS-GNPs.

**Table 3:** PK parameters of IT-NPs.

Types of NPs	Route	AUC	C_max_	T_max_	T_1/2_	References
CBD-NE	IT	3.7-fold higher	210 ng/g	120 min	NA	[109]
CBD-PCNPs	IT	NA	94 ng/g	30 min	NA	[109]
Liposomal Cytarabine	IT	NA	NA	NA	43 h	[25,26]
IMI/CIL (API)	IT	NA	4.5 µg/100 mg	2h	8 h	[124]
IMI/CIL (API)	IV	NA	1.5 µg/100 mg	2h	<8 h	[124]

Abbreviations: Cannabidiol: CBD, Half-life: T_1/2_, Area under curve: AUC, Maximum concentration: C_max_, Maximum time: T_max_, Imipenem/cilastatin: IMI/CIL.

**Table 4:** Clinical IT-NP application targeting CNS parenchyma in CNS diseases.

Nanoparticle Type	Drug	Disease	Delivery Route	Phase	Size (nm)	Surface-Modification	Penetration Efficiency (%)	References
Lipid-based NPs (liposome)	DepoCyte^®^	Brain metastases	IT	Phase I	NA	NA	NA	NCT00854867
	DepoCyte^®^	Meningeal metastasis of breast cancer	IT	Phase III	NA	NA	NA	NCT01645839
	Depocyt (Cytarabine)	Meningeal neoplasms	IT	Phase IV	NA	NA	NA	NCT00029523
	Depocyt (Cytarabine)	CNS metastases tumors	IT	Phase II	NA	NA	NA	NCT00992602
Polymeric-NPs	Carmustine wafer	Glioblastoma	SI	Phase I/II	NA	NA	NA	NCT00984438
	Carmustine implants	Brain metastases	SI	Phase II	NA	NA	NA	NCT00003878
Inorganic NPs	-	-	-	-	-	-	-	-
Cell-derived biomimetic NPs	Exosomes	CVD	Intraparenchymal	Phase I/II	NA	NA	NA	NCT03384433
	Stem cells (MSCs)	Multiple sclerosis	IT/IV	Phase II	NA	NA	NA	NCT02166021
	Stem cells (HSCs)	Multiple sclerosis	IT/IV	Phase III	NA	NA	NA	NCT04047628

Abbreviations: Surgical Implant: SI, Intrathecal: IT, Cerebrovascular disease: CVD, Not available: NA.

**Table 5:** LRPs in BBB and BCSFB.

LRPs	BBB	BCSFB	References
LRP1	Debated	Consistently expressed	[253,254,255,256,257]
LRP2	Not expressed	Highly expressed	[224,263]
LRP8	Expressed in development	Highly expressed	[264,265]

Abbreviations: LRPs: LDL receptor-related protein.

**Table 6:** BCSFB pathways or transporters and receptors for drug delivery.

Receptor	Endogenous Substrate	Evidence of Transcytosis Across CP	Clinical Relevance	References
Transferrin Receptor (TfR1)	Transferrin	Indirect	Treatment of iron-related disorders, neurodegenerative diseases	[235,236,237,278]
Insulin Receptor (IR)	Insulin	Limited data	Regulating glucose homeostasis	[247]
LDL Receptor	LDL	Limited data	Cholesterol transport	[253,278]
LRP1	Various (α2-macroglobulin, matrix metalloproteases, different carrier proteins…)	Indirect	Cholesterol transport	[253]
LRP2	Partial overlapping substrate specificity with LRP1, Morphogenic factors, leptin, insulin-like growth factor I (developmental relevance)	Yes, for endogenous substrate	Cholesterol	[224,263]
			transport	[264,265]
LRP8	Partial overlapping substrate specificity with LRP1, Selenoprotein P	Partial, indirect	Cholesterol	[224,263]
			transport	[264,265]
Folate Receptor (FRα)	Folate	Yes, for endogenous substrate, through exosome	Cancer therapy, neurological disorders	[267,268]
Unknown (possibly SPARC, Glycophorins)	Plasma proteins (albumin, fetuin, hemopexin, α-fetoprotein)	Yes, for exogenous proteins	Potential for targeted drug delivery, biomarker discovery	[279]

Abbreviations: Choroid plexus, CP; lipoprotein receptor-related protein, LRP.

## Data Availability

Data sharing is not applicable.

## List of Abbreviations

5-HT	Serotonin
5MTHF	5-methyltetrahydrofolate
ApoA	Apolipoprotein A
ApoE	Apolipoprotein E
AMT	Absorptive-Mediated Transcytosis
API	Active Pharmaceutical Ingredient
AUC	Area Under the Curve
Au198	Radioactive Gold-198
BACE1	Beta-Secretase 1
BCSFB	Blood–Cerebrospinal Fluid Barrier (also known as Blood–CSF Barrier)
CBD	Cannabidiol
CMT	Solute Carrier-Mediated Transcytosis
CNS	Central Nervous System
CPP	Cell-Penetrating Peptide
CPPs	Cell-Penetrating Peptides
CP	Choroid Plexus
CPECs	Choroid Plexus Epithelial Cells
CSF	Cerebrospinal Fluid
DDS	Drug Delivery Systems
DiD	A hydrophobic carbocyanine dye
DOX	Doxorubicin
ECs	Endothelial Cells
EG-1962	PLGA-Encapsulated Nimodipine
EXPAREL^®^	Liposomal Bupivacaine
FR	Folate Receptor
FRα	Folate Receptor Alpha
GLUT	Glucose Transporter
HIRMAb	Human Insulin Receptor Monoclonal Antibody
ICM	Intracisternal Magna
IMI/CIL	Imipenem/Cilastatin
IT	Intrathecal
IT-NPs	Intrathecal Nanoparticles
IVT	Intraventricular
LRP1	LDL Receptor-Related Protein 1
LDL	Low-Density Lipoprotein
LDLR	Low-Density Lipoprotein Receptor
L-DOPA	Levodopa
LPEI	Linear Polyethyleneimine
MPSI	Mucopolysaccharidosis Type I
NPs	Nanoparticles
PCL	Polycaprolactone
PCL-NPs	Polycaprolactone Nanoparticles
PCNPs	Polymer-Coated Nanoparticles
PEG	Polyethylene Glycol
PEI	Polyethyleneimine
PK	Pharmacokinetic
PKs	Pharmacokinetics
PDs	Pharmacodynamics
PLGA	Poly(lactic-co-glycolic acid)
PNPs	Polymeric Nanoparticles
RMT	Receptor-Mediated Transcytosis
RCF	Reduced Folate Carrier
SAS	Subarachnoid Space
SLNPs	Solid Lipid Nanoparticles
siRNA	Small Interfering RNA
TAT	Trans-Activator of Transcription
TfR1	Transferrin Receptor 1
VSOP-C184	Very Small Superparamagnetic Iron Oxide Particle C184
ZO	Zonula Occludens

## References

[1] Solár P., Zamani A., Kubíčková L., Dubový P., Joukal M. (2020). Choroid plexus and the blood-cerebrospinal fluid barrier in disease. Fluids Barriers CNS.

[2] Dabbagh F., Schroten H., Schwerk C. (2022). In Vitro Models of the Blood-Cerebrospinal Fluid Barrier and Their Applications in the Development and Research of (Neuro)Pharmaceuticals. Pharmaceutics.

[3] Begley D.J. (2004). Delivery of therapeutic agents to the central nervous system: The problems and the possibilities. Pharmacol. Ther..

[4] Fowler M.J., Cotter J.D., Knight B.E., Sevick-Muraca E.M., Sandberg D.I., Sirianni R.W. (2020). Intrathecal drug delivery in the era of nanomedicine. Adv. Drug Deliv. Rev..

[5] Stielow M., Witczyńska A., Kubryń N., Fijałkowski Ł., Nowaczyk J., Nowaczyk A. (2023). The Bioavailability of Drugs—The Current State of Knowledge. Molecules.

[6] Wang F., Yang G., Zhou Y., Song H., Xiong L., Wang L., Shen X. (2022). Pharmacokinetics of niazirin from Moringa oleifera Lam in rats by UPLC-MS/MS: Absolute bioavailability and dose proportionality. eFood.

[7] Pardridge W.M. (2005). The blood-brain barrier: Bottleneck in brain drug development. NeuroRx.

[8] Pardridge W.M. (1998). CNS drug design based on principles of blood-brain barrier transport. J. Neurochem..

[9] Cresswell F.V., Meya D.B., Kagimu E., Grint D., Te Brake L., Kasibante J., Martyn E., Rutakingirwa M., Quinn C.M., Okirwoth M. (2021). High-dose oral and intravenous rifampicin for the treatment of tuberculous meningitis in predominantly human immunodeficiency virus (HIV)-positive Ugandan adults: A phase II open-label randomized controlled trial. Clin. Infect. Dis..

[10] Calias P., Banks W.A., Begley D., Scarpa M., Dickson P. (2014). Intrathecal delivery of protein therapeutics to the brain: A critical reassessment. Pharmacol. Ther..

[11] Kung Y., Chen K.Y., Liao W.H., Hsu Y.H., Wu C.H., Hsiao M.Y., Huang A.P., Chen W.S. (2022). Facilitating drug delivery in the central nervous system by opening the blood-cerebrospinal fluid barrier with a single low energy shockwave pulse. Fluids Barriers CNS.

[12] Cho S.R. (2023). Intrathecal Baclofen Therapy: Pros and Cons. Ann. Rehabil. Med..

[13] Kang X., Chen F., Yang S.B., Wang Y.L., Qian Z.H., Li Y., Lin H., Li P., Peng Y.C., Wang X.M. (2022). Intrathecal methotrexate in combination with systemic chemotherapy in glioblastoma patients with leptomeningeal dissemination: A retrospective analysis. World J. Clin. Cases.

[14] Berg S.L., Poplack D.G. (1996). Treatment of Meningeal Malignancy. Oncologist.

[15] Sandberg D.I., Solano J., Petito C.K., Mian A., Mou C., Koru-Sengul T., Gonzalez-Brito M., Padgett K.R., Luqman A., Buitrago J.C. (2010). Safety and pharmacokinetic analysis of methotrexate administered directly into the fourth ventricle in a piglet model. J. Neurooncol..

[16] Beck M., Flachenecker P., Magnus T., Giess R., Reiners K., Toyka K.V., Naumann M. (2005). Autonomic dysfunction in ALS: A preliminary study on the effects of intrathecal BDNF. Amyotroph. Lateral Scler. Other Mot. Neuron. Disord..

[17] Shapiro W., Schmid M., Glantz M., Miller J. (2006). A randomized phase III/IV study to determine benefit and safety of cytarabine liposome injection for treatment of neoplastic meningitis. J. Clin. Oncol..

[18] Papisov M.I., Belov V.V., Gannon K.S. (2013). Physiology of the intrathecal bolus: The leptomeningeal route for macromolecule and particle delivery to CNS. Mol. Pharm..

[19] Glantz M.J., LaFollette S., Jaeckle K.A., Shapiro W., Swinnen L., Rozental J.R., Phuphanich S., Rogers L.R., Gutheil J.C., Batchelor T. (1999). Randomized trial of a slow-release versus a standard formulation of cytarabine for the intrathecal treatment of lymphomatous meningitis. J. Clin. Oncol..

[20] Zimm S., Collins J.M., Miser J., Chatterji D., Poplack D.G. (1984). Cytosine arabinoside cerebrospinal fluid kinetics. Clin. Pharmacol. Ther..

[21] Wolf D.A., Hesterman J.Y., Sullivan J.M., Orcutt K.D., Silva M.D., Lobo M., Wellman T., Hoppin J., Verma A. (2016). Dynamic dual-isotope molecular imaging elucidates principles for optimizing intrathecal drug delivery. JCI Insight.

[22] Flack S.H., Bernards C.M. (2010). Cerebrospinal fluid and spinal cord distribution of hyperbaric bupivacaine and baclofen during slow intrathecal infusion in pigs. Anesthesiology.

[23] Kitamura I., Kochi M., Matsumoto Y., Ueoka R., Kuratsu J., Ushio Y. (1996). Intrathecal chemotherapy with 1,3-bis(2-chloroethyl)-1-nitrosourea encapsulated into hybrid liposomes for meningeal gliomatosis: An experimental study. Cancer Res..

[24] Householder K.T., DiPerna D.M., Chung E.P., Luning A.R., Nguyen D.T., Stabenfeldt S.E., Mehta S., Sirianni R.W. (2018). pH driven precipitation of quisinostat onto PLA-PEG nanoparticles enables treatment of intracranial glioblastoma. Colloids Surf. B Biointerfaces.

[25] Peyrl A., Sauermann R., Traunmueller F., Azizi A.A., Gruber-Olipitz M., Gupper A., Slavc I. (2009). Pharmacokinetics and safety of intrathecal liposomal cytarabine in children aged < 3 years. Clin. Pharmacokinet..

[26] Peyrl A., Sauermann R., Chocholous M., Azizi A.A., Jäger W., Höferl M., Slavc I. (2014). Pharmacokinetics and toxicity of intrathecal liposomal cytarabine in children and adolescents following age-adapted dosing. Clin. Pharmacokinet..

[27] Swami A., Shi J., Gadde S., Votruba A.R., Kolishetti N., Farokhzad O.C., Svenson S., Prud’homme R.K. (2012). Nanoparticles for Targeted and Temporally Controlled Drug Delivery. Multifunctional Nanoparticles for Drug Delivery Applications: Imaging, Targeting, and Delivery.

[28] Householder K.T., Dharmaraj S., Sandberg D.I., Wechsler-Reya R.J., Sirianni R.W. (2019). Fate of nanoparticles in the central nervous system after intrathecal injection in healthy mice. Sci. Rep..

[29] Bhojwani D., Pui C.H. (2008). Intrathecal liposomal cytarabine: More friend than foe?. Leuk. Lymphoma.

[30] Moreno L., García Ariza M.A., Cruz O., Calvo C., Fuster J.L., Salinas J.A., Moscardo C., Portugal R., Merino J.M., Madero L. (2016). Liposomal cytarabine for the treatment of leptomeningeal dissemination of central nervous system tumours in children and adolescents. Pediatría.

[31] Spina M., Chimienti E., Martellotta F., Vaccher E., Berretta M., Zanet E., Lleshi A., Canzonieri V., Bulian P., Tirelli U. (2010). Phase 2 study of intrathecal, long-acting liposomal cytarabine in the prophylaxis of lymphomatous meningitis in human immunodeficiency virus-related non-Hodgkin lymphoma. Cancer.

[32] Kim S., Kim D.J., Geyer M.A., Howell S.B. (1987). Multivesicular liposomes containing 1-beta-D-arabinofuranosylcytosine for slow-release intrathecal therapy. Cancer Res..

[33] Lilius T.O., Mortensen K.N., Deville C., Lohela T.J., Stæger F.F., Sigurdsson B., Fiordaliso E.M., Rosenholm M., Kamphuis C., Beekman F.J. (2023). Glymphatic-assisted perivascular brain delivery of intrathecal small gold nanoparticles. J. Control Release.

[34] Rudick R.A., Zirretta D.K., Herndon R.M. (1982). Clearance of albumin from mouse subarachnoid space: A measure of CSF bulk flow. J. Neurosci. Methods.

[35] Simon M.J., Iliff J.J. (2016). Regulation of cerebrospinal fluid (CSF) flow in neurodegenerative, neurovascular and neuroinflammatory disease. Biochim. Biophys. Acta.

[36] Liu R., Jia W., Wang Y., Hu C., Yu W., Huang Y., Wang L., Gao H. (2022). Glymphatic System and Subsidiary Pathways Drive Nanoparticles Away from the Brain. Research.

[37] Sharma H.S. (2003). Blood-Spinal Cord and Brain Barriers in Health and Disease.

[38] Redzic Z.B. (2013). Studies on the human choroid plexus in vitro. Fluids Barriers CNS.

[39] Tumani H., Huss A., Bachhuber F. (2018). The cerebrospinal fluid and barriers–anatomic and physiologic considerations. Handb. Clin. Neurol..

[40] Brightman M.W., Reese T.S. (1969). Junctions between intimately apposed cell membranes in the vertebrate brain. J. Cell Biol..

[41] Rall D.P. (1968). Transport through the ependymal linings. Prog. Brain Res..

[42] Redzic Z.B., Segal M.B. (2004). The structure of the choroid plexus and the physiology of the choroid plexus epithelium. Adv. Drug Deliv. Rev..

[43] Zheng W., Zhao Q. (2002). The blood-CSF barrier in culture. Development of a primary culture and transepithelial transport model from choroidal epithelial cells. Methods Mol. Biol..

[44] Nilsson C., Lindvall-Axelsson M., Owman C. (1992). Neuroendocrine regulatory mechanisms in the choroid plexus-cerebrospinal fluid system. Brain Res. Brain Res. Rev..

[45] Ueno M., Chiba Y., Murakami R., Matsumoto K., Kawauchi M., Fujihara R. (2016). Blood-brain barrier and blood-cerebrospinal fluid barrier in normal and pathological conditions. Brain Tumor Pathol..

[46] Saunders N.R., Daneman R., Dziegielewska K.M., Liddelow S.A. (2013). Transporters of the blood-brain and blood-CSF interfaces in development and in the adult. Mol. Asp. Med..

[47] Wright E.M. (1977). Effect of bicarbonate and other buffers on choroid plexus Na+/K+pump. Biochim. Biophys. Acta.

[48] Engelhardt B., Sorokin L. (2009). The blood-brain and the blood-cerebrospinal fluid barriers: Function and dysfunction. Semin. Immunopathol..

[49] Hou Z., Matherly L.H. (2014). Biology of the major facilitative folate transporters SLC19A1 and SLC46A1. Curr. Top. Membr..

[50] Adinolfi M., Beck S.E., Haddad S.A., Seller M.J. (1976). Permeability of the blood-cerebrospinal fluid barrier to plasma proteins during foetal and perinatal life. Nature.

[51] Spector R., Johanson C.E. (1989). The mammalian choroid plexus. Sci. Am..

[52] Groothuis D.R., Levy R.M. (1997). The entry of antiviral and antiretroviral drugs into the central nervous system. J. Neurovirology.

[53] Segal M.B. (2000). The choroid plexuses and the barriers between the blood and the cerebrospinal fluid. Cell. Mol. Neurobiol..

[54] Enting R.H., Hoetelmans R.M., Lange J.M., Burger D.M., Beijnen J.H., Portegies P. (1998). Antiretroviral drugs and the central nervous system. Aids.

[55] Davson H., Hollingsworth G., Segal M. (1970). The mechanism of drainage of the cerebrospinal fluid. Brain.

[56] Saunders N., Habgood M., Dziegielewska K. (1999). Barrier mechanisms in the brain, I. Adult brain. Clin. Exp. Pharmacol. Physiol..

[57] Garner C., Brown P.D. (1992). Two types of chloride channel in the apical membrane of rat choroid plexus epithelial cells. Brain Res..

[58] de Lange E.C. (2004). Potential role of ABC transporters as a detoxification system at the blood-CSF barrier. Adv. Drug Deliv. Rev..

[59] Chen X., Keep R.F., Liang Y., Zhu H.J., Hammarlund-Udenaes M., Hu Y., Smith D.E. (2017). Influence of peptide transporter 2 (PEPT2) on the distribution of cefadroxil in mouse brain: A microdialysis study. Biochem. Pharmacol..

[60] Sarin H. (2010). Physiologic upper limits of pore size of different blood capillary types and another perspective on the dual pore theory of microvascular permeability. J. Angiogenes Res..

[61] Strazielle N., Ghersi-Egea J.F. (2000). Choroid plexus in the central nervous system: Biology and physiopathology. J. Neuropathol. Exp. Neurol..

[62] Carpenter S.J., McCarthy L.E., Borison H.L. (1970). Electron microscopic study of the epiplexus (Kolmer) cells of the cat choroid plexus. Z. Zellforsch. Mikrosk. Anat..

[63] Hosoya Y., Fujita T. (1973). Scanning electron microscope observation of intraventricular macrophages (Kolmer cells) in the rat brain. Arch. Histol. Jpn..

[64] Maslieieva V., Thompson R.J. (2014). A critical role for pannexin-1 in activation of innate immune cells of the choroid plexus. Channels.

[65] Quintela T., Albuquerque T., Lundkvist G., Carmine Belin A., Talhada D., Gonçalves I., Carro E., Santos C.R.A. (2018). The choroid plexus harbors a circadian oscillator modulated by estrogens. Chronobiol. Int..

[66] Santos C.R.A., Duarte A.C., Costa A.R., Tomás J., Quintela T., Gonçalves I. (2019). The senses of the choroid plexus. Prog. Neurobiol..

[67] Fishman R.A. (1966). Blood-brain and CSF barriers to penicillin and related organic acids. Arch. Neurol..

[68] Bode U., Magrath I.T., Bleyer W.A., Poplack D.G., Glaubiger D.L. (1980). Active transport of methotrexate from cerebrospinal fluid in humans. Cancer Res..

[69] Del Bigio M.R. (1995). The ependyma: A protective barrier between brain and cerebrospinal fluid. Glia.

[70] Nicholson C. (1999). Signals that go with the flow. Trends Neurosci..

[71] Gotow T., Hashimoto P.H. (1980). Fine structure of ependymal cysts in and around the area postrema of the rat. Cell Tissue Res..

[72] Whish S., Dziegielewska K.M., Møllgård K., Noor N.M., Liddelow S.A., Habgood M.D., Richardson S.J., Saunders N.R. (2015). The inner CSF-brain barrier: Developmentally controlled access to the brain via intercellular junctions. Front. Neurosci..

[73] Brightman M.W. (1968). The intracerebral movement of proteins injected into blood and cerebrospinal fluid of mice. Prog. Brain Res..

[74] Drake R.L. (2005). Gray’s Anatomy for Students.

[75] Crossman A.R., Neary D. (2018). Neuroanatomy E-Book: An Illustrated Colour Text.

[76] Wolburg H., Wolburg-Buchholz K., Liebner S., Engelhardt B. (2001). Claudin-1, claudin-2 and claudin-11 are present in tight junctions of choroid plexus epithelium of the mouse. Neurosci. Lett..

[77] Brightman M., Palay S. (1963). The fine structure of ependyma in the brain of the rat. J. Cell Biol..

[78] Pappas G.D., Tennyson V.M. (1962). An electron microscopic study of the passage of colloidal particles from the blood vessels of the ciliary processes and choroid plexus of the rabbit. J. Cell Biol..

[79] Menheniott T.R., Charalambous M., Ward A. (2010). Derivation of primary choroid plexus epithelial cells from the mouse. Methods Mol. Biol..

[80] Drulis-Kawa Z., Dorotkiewicz-Jach A. (2010). Liposomes as delivery systems for antibiotics. Int. J. Pharm..

[81] Underwood C., Van Eps A. (2012). Nanomedicine and veterinary science: The reality and the practicality. Vet. J..

[82] Peviani M., Capasso Palmiero U., Cecere F., Milazzo R., Moscatelli D., Biffi A. (2019). Biodegradable polymeric nanoparticles administered in the cerebrospinal fluid: Brain biodistribution, preferential internalization in microglia and implications for cell-selective drug release. Biomaterials.

[83] Betzer O., Shilo M., Opochinsky R., Barnoy E., Motiei M., Okun E., Yadid G., Popovtzer R. (2017). The effect of nanoparticle size on the ability to cross the blood-brain barrier: An in vivo study. Nanomedicine.

[84] Adson A., Raub T.J., Burton P.S., Barsuhn C.L., Hilgers A.R., Audus K.L., Ho N.F. (1994). Quantitative approaches to delineate paracellular diffusion in cultured epithelial cell monolayers. J. Pharm. Sci..

[85] Jo D.H., Kim J.H., Lee T.G., Kim J.H. (2015). Size, surface charge, and shape determine therapeutic effects of nanoparticles on brain and retinal diseases. Nanomedicine.

[86] Chithrani B.D., Ghazani A.A., Chan W.C. (2006). Determining the size and shape dependence of gold nanoparticle uptake into mammalian cells. Nano Lett..

[87] Hillaireau H., Couvreur P. (2009). Nanocarriers’ entry into the cell: Relevance to drug delivery. Cell. Mol. Life Sci..

[88] Schipper M.L., Iyer G., Koh A.L., Cheng Z., Ebenstein Y., Aharoni A., Keren S., Bentolila L.A., Li J., Rao J. (2009). Particle size, surface coating, and PEGylation influence the biodistribution of quantum dots in living mice. Small.

[89] Fytianos K., Chortarea S., Rodriguez-Lorenzo L., Blank F., von Garnier C., Petri-Fink A., Rothen-Rutishauser B. (2017). Aerosol Delivery of Functionalized Gold Nanoparticles Target and Activate Dendritic Cells in a 3D Lung Cellular Model. ACS Nano.

[90] Zhang L., Becton M., Wang X. (2015). Designing nanoparticle translocation through cell membranes by varying amphiphilic polymer coatings. J. Phys. Chem. B.

[91] Moyano D.F., Goldsmith M., Solfiell D.J., Landesman-Milo D., Miranda O.R., Peer D., Rotello V.M. (2012). Nanoparticle hydrophobicity dictates immune response. J. Am. Chem. Soc..

[92] Zylberberg C., Gaskill K., Pasley S., Matosevic S. (2017). Engineering liposomal nanoparticles for targeted gene therapy. Gene Ther..

[93] Liu Y., An S., Li J., Kuang Y., He X., Guo Y., Ma H., Zhang Y., Ji B., Jiang C. (2016). Brain-targeted co-delivery of therapeutic gene and peptide by multifunctional nanoparticles in Alzheimer’s disease mice. Biomaterials.

[94] Nowak M., Brown T.D., Graham A., Helgeson M.E., Mitragotri S. (2020). Size, shape, and flexibility influence nanoparticle transport across brain endothelium under flow. Bioeng. Transl. Med..

[95] Arnida, Janát-Amsbury M.M., Ray A., Peterson C.M., Ghandehari H. (2011). Geometry and surface characteristics of gold nanoparticles influence their biodistribution and uptake by macrophages. Eur. J. Pharm. Biopharm..

[96] Kim B., Han G., Toley B.J., Kim C.K., Rotello V.M., Forbes N.S. (2010). Tuning payload delivery in tumour cylindroids using gold nanoparticles. Nat. Nanotechnol..

[97] Toro R., Perron M., Pike B., Richer L., Veillette S., Pausova Z., Paus T. (2008). Brain size and folding of the human cerebral cortex. Cereb. Cortex.

[98] Mestre H., Tithof J., Du T., Song W., Peng W., Sweeney A.M., Olveda G., Thomas J.H., Nedergaard M., Kelley D.H. (2018). Flow of cerebrospinal fluid is driven by arterial pulsations and is reduced in hypertension. Nat. Commun..

[99] Barua S., Mitragotri S. (2014). Challenges associated with penetration of nanoparticles across cell and tissue barriers: A review of current status and future prospects. Nano Today.

[100] Varela J.A., Dupuis J.P., Etchepare L., Espana A., Cognet L., Groc L. (2016). Targeting neurotransmitter receptors with nanoparticles in vivo allows single-molecule tracking in acute brain slices. Nat. Commun..

[101] Albertazzi L., Gherardini L., Brondi M., Sulis Sato S., Bifone A., Pizzorusso T., Ratto G.M., Bardi G. (2013). In vivo distribution and toxicity of PAMAM dendrimers in the central nervous system depend on their surface chemistry. Mol. Pharm..

[102] Kim H., Choi B., Lim H., Min H., Oh J.H., Choi S., Cho J.G., Park J.S., Lee S.J. (2017). Polyamidoamine dendrimer-conjugated triamcinolone acetonide attenuates nerve injury-induced spinal cord microglia activation and mechanical allodynia. Mol. Pain..

[103] Dai H., Navath R.S., Balakrishnan B., Guru B.R., Mishra M.K., Romero R., Kannan R.M., Kannan S. (2010). Intrinsic targeting of inflammatory cells in the brain by polyamidoamine dendrimers upon subarachnoid administration. Nanomedicine.

[104] Mundt A.P., Winter C., Mueller S., Wuerfel J., Tysiak E., Schnorr J., Taupitz M., Heinz A., Juckel G. (2009). Targeting activated microglia in Alzheimer’s pathology by intraventricular delivery of a phagocytosable MRI contrast agent in APP23 transgenic mice. Neuroimage.

[105] Rieselbach R.E., Chiro G.D., Freireich E.J., Rall D.P. (1962). Subarachnoid distribution of drugs after lumbar injection. N. Engl. J. Med..

[106] Sumner J.P., Shapiro E.M., Maric D., Conroy R., Koretsky A.P. (2009). In vivo labeling of adult neural progenitors for MRI with micron sized particles of iron oxide: Quantification of labeled cell phenotype. Neuroimage.

[107] Liu C.H., Ren J.Q., Yang J., Liu C.M., Mandeville J.B., Rosen B.R., Bhide P.G., Yanagawa Y., Liu P.K. (2009). DNA-based MRI probes for specific detection of chronic exposure to amphetamine in living brains. J. Neurosci..

[108] Bharali D.J., Klejbor I., Stachowiak E.K., Dutta P., Roy I., Kaur N., Bergey E.J., Prasad P.N., Stachowiak M.K. (2005). Organically modified silica nanoparticles: A nonviral vector for in vivo gene delivery and expression in the brain. Proc. Natl. Acad. Sci. USA.

[109] Muresan P., Woodhams S., Smith F., Taresco V., Shah J., Wong M., Chapman V., Smith S., Hathway G., Rahman R. (2023). Evaluation of cannabidiol nanoparticles and nanoemulsion biodistribution in the central nervous system after intrathecal administration for the treatment of pain. Nanomed. Nanotechnol. Biol. Med..

[110] Hagihara Y., Saitoh Y., Kaneda Y., Kohmura E., Yoshimine T. (2000). Widespread gene transfection into the central nervous system of primates. Gene Ther..

[111] Kimelberg H.K., Tracy T.F., Watson R.E., Kung D., Reiss F.L., Bourke R.S. (1978). Distribution of free and liposome-entrapped [3H]methotrexate in the central nervous system after intracerebroventricular injection in a primate. Cancer Res..

[112] Anderson D.M., Hall L.L., Ayyalapu A.R., Irion V.R., Nantz M.H., Hecker J.G. (2003). Stability of mRNA/cationic lipid lipoplexes in human and rat cerebrospinal fluid: Methods and evidence for nonviral mRNA gene delivery to the central nervous system. Hum. Gene Ther..

[113] Wood R.I., Johnson L.R., Chu L., Schad C., Self D.W. (2004). Testosterone reinforcement: Intravenous and intracerebroventricular self-administration in male rats and hamsters. Psychopharmacology.

[114] Frye C.A., Duncan J.E. (1996). Estradiol benzoate potentiates neuroactive steroids’ effects on pain sensitivity. Pharmacol. Biochem. Behav..

[115] Vaz G.C., Bahia A.P., de Figueiredo Müller-Ribeiro F.C., Xavier C.H., Patel K.P., Santos R.A., Moreira F.A., Frézard F., Fontes M.A. (2015). Cardiovascular and behavioral effects produced by administration of liposome-entrapped GABA into the rat central nervous system. Neuroscience.

[116] Shtein L., Toker L., Bersudsky Y., Belmaker R.H., Agam G. (2013). The inositol monophosphatase inhibitor L-690,330 affects pilocarpine-behavior and the forced swim test. Psychopharmacology.

[117] Neelov I.M., Janaszewska A., Klajnert B., Bryszewska M., Makova N.Z., Hicks D., Pearson H.A., Vlasov G.P., Ilyash M.Y., Vasilev D.S. (2013). Molecular properties of lysine dendrimers and their interactions with Aβ-peptides and neuronal cells. Curr. Med. Chem..

[118] Jankowski W., Strosznajder J. (1992). Uptake and subcellular distribution of intraventricularly injected [1-3H]dolichol in rat brain. Acta Biochim. Pol..

[119] Rodríguez G., Soria G., Coll E., Rubio L., Barbosa-Barros L., López-Iglesias C., Planas A.M., Estelrich J., de la Maza A., López O. (2010). Bicosomes: Bicelles in dilute systems. Biophys. J..

[120] Akita H., Nakatani T., Kuroki K., Maenaka K., Tange K., Nakai Y., Harashima H. (2015). Effect of hydrophobic scaffold on the cellular uptake and gene transfection activities of DNA-encapsulating liposomal nanoparticles via intracerebroventricular administration. Int. J. Pharm..

[121] Helmschrodt C., Höbel S., Schöniger S., Bauer A., Bonicelli J., Gringmuth M., Fietz S.A., Aigner A., Richter A., Richter F. (2017). Polyethylenimine Nanoparticle-Mediated siRNA Delivery to Reduce α-Synuclein Expression in a Model of Parkinson’s Disease. Mol. Ther. Nucleic Acids.

[122] Shyam R., Ren Y., Lee J., Braunstein K.E., Mao H.Q., Wong P.C. (2015). Intraventricular Delivery of siRNA Nanoparticles to the Central Nervous System. Mol. Ther. Nucleic Acids.

[123] Goula D., Remy J.S., Erbacher P., Wasowicz M., Levi G., Abdallah B., Demeneix B.A. (1998). Size, diffusibility and transfection performance of linear PEI/DNA complexes in the mouse central nervous system. Gene Ther..

[124] Wang Y., Qiu L., Dong J., Wang B., Shi Z., Liu B., Wang W., Zhang J., Cai S., Ye G. (2013). Comparison of the pharmacokinetics of imipenem after intravenous and intrathecal administration in rabbits. Eur. Rev. Med. Pharmacol. Sci..

[125] Strazielle N., Ghersi-Egea J.F. (2016). Potential Pathways for CNS Drug Delivery Across the Blood-Cerebrospinal Fluid Barrier. Curr. Pharm. Des..

[126] Abdul Razzak R., Florence G.J., Gunn-Moore F.J. (2019). Approaches to CNS Drug Delivery with a Focus on Transporter-Mediated Transcytosis. Int. J. Mol. Sci..

[127] Broadwell R.D., Baker-Cairns B.J., Friden P.M., Oliver C., Villegas J.C. (1996). Transcytosis of protein through the mammalian cerebral epithelium and endothelium. III. Receptor-mediated transcytosis through the blood-brain barrier of blood-borne transferrin and antibody against the transferrin receptor. Exp. Neurol..

[128] Thom G., Burrell M., Haqqani A.S., Yogi A., Lessard E., Brunette E., Delaney C., Baumann E., Callaghan D., Rodrigo N. (2018). Enhanced Delivery of Galanin Conjugates to the Brain through Bioengineering of the Anti-Transferrin Receptor Antibody OX26. Mol. Pharm..

[129] Boado R.J., Hui E.K., Lu J.Z., Pardridge W.M. (2010). Drug targeting of erythropoietin across the primate blood-brain barrier with an IgG molecular Trojan horse. J. Pharmacol. Exp. Ther..

[130] Boado R.J., Zhang Y., Zhang Y., Pardridge W.M. (2007). Humanization of anti-human insulin receptor antibody for drug targeting across the human blood-brain barrier. Biotechnol. Bioeng..

[131] Giugliani R., Giugliani L., de Oliveira Poswar F., Donis K.C., Corte A.D., Schmidt M., Boado R.J., Nestrasil I., Nguyen C., Chen S. (2018). Neurocognitive and somatic stabilization in pediatric patients with severe Mucopolysaccharidosis Type I after 52 weeks of intravenous brain-penetrating insulin receptor antibody-iduronidase fusion protein (valanafusp alpha): An open label phase 1-2 trial. Orphanet J. Rare Dis..

[132] Bryniarski M.A., Ren T., Rizvi A.R., Snyder A.M., Morris M.E. (2020). Targeting the Choroid Plexuses for Protein Drug Delivery. Pharmaceutics.

[133] Shu C., Shen H., Teuscher N.S., Lorenzi P.J., Keep R.F., Smith D.E. (2002). Role of PEPT2 in peptide/mimetic trafficking at the blood-cerebrospinal fluid barrier: Studies in rat choroid plexus epithelial cells in primary culture. J. Pharmacol. Exp. Ther..

[134] Joseph A., Nance E. (2022). Nanotherapeutics and the Brain. Annu. Rev. Chem. Biomol. Eng..

[135] Sharma M., Dube T., Chibh S., Kour A., Mishra J., Panda J.J. (2019). Nanotheranostics, a future remedy of neurological disorders. Expert Opin. Drug Deliv..

[136] Dong X. (2018). Current Strategies for Brain Drug Delivery. Theranostics.

[137] Kim J., Ahn S.I., Kim Y. (2019). Nanotherapeutics Engineered to Cross the Blood-Brain Barrier for Advanced Drug Delivery to the Central Nervous System. J. Ind. Eng. Chem..

[138] Tang W., Fan W., Lau J., Deng L., Shen Z., Chen X. (2019). Emerging blood-brain-barrier-crossing nanotechnology for brain cancer theranostics. Chem. Soc. Rev..

[139] Tang L., Feng Y., Gao S., Mu Q., Liu C. (2021). Nanotherapeutics Overcoming the Blood-Brain Barrier for Glioblastoma Treatment. Front. Pharmacol..

[140] Alotaibi B.S., Buabeid M., Ibrahim N.A., Kharaba Z.J., Ijaz M., Noreen S., Murtaza G. (2021). Potential of Nanocarrier-Based Drug Delivery Systems for Brain Targeting: A Current Review of Literature. Int. J. Nanomed..

[141] Nehra M., Uthappa U.T., Kumar V., Kumar R., Dixit C., Dilbaghi N., Mishra Y.K., Kumar S., Kaushik A. (2021). Nanobiotechnology-assisted therapies to manage brain cancer in personalized manner. J. Control Release.

[142] Furtado D., Björnmalm M., Ayton S., Bush A.I., Kempe K., Caruso F. (2018). Overcoming the Blood-Brain Barrier: The Role of Nanomaterials in Treating Neurological Diseases. Adv. Mater..

[143] Lynch M.J., Gobbo O.L. (2021). Advances in Non-Animal Testing Approaches towards Accelerated Clinical Translation of Novel Nanotheranostic Therapeutics for Central Nervous System Disorders. Nanomaterials.

[144] Kumar A., Chaudhary R.K., Singh R., Singh S.P., Wang S.Y., Hoe Z.Y., Pan C.T., Shiue Y.L., Wei D.Q., Kaushik A.C. (2020). Nanotheranostic Applications for Detection and Targeting Neurodegenerative Diseases. Front. Neurosci..

[145] Wong H.L., Wu X.Y., Bendayan R. (2012). Nanotechnological advances for the delivery of CNS therapeutics. Adv. Drug Deliv. Rev..

[146] De Boer A., Van Der Sandt I., Gaillard P. (2003). The role of drug transporters at the blood-brain barrier. Annu. Rev. Pharmacol. Toxicol..

[147] Huwyler J., Wu D., Pardridge W.M. (1996). Brain drug delivery of small molecules using immunoliposomes. Proc. Natl. Acad. Sci. USA.

[148] Huwyler J., Yang J., Pardridge W.M. (1997). Receptor mediated delivery of daunomycin using immunoliposomes: Pharmacokinetics and tissue distribution in the rat. J. Pharmacol. Exp. Ther..

[149] Zhang Y., Calon F., Zhu C., Boado R.J., Pardridge W.M. (2003). Intravenous nonviral gene therapy causes normalization of striatal tyrosine hydroxylase and reversal of motor impairment in experimental parkinsonism. Hum. Gene Ther..

[150] Pang Z., Lu W., Gao H., Hu K., Chen J., Zhang C., Gao X., Jiang X., Zhu C. (2008). Preparation and brain delivery property of biodegradable polymersomes conjugated with OX26. J. Control. Release.

[151] Shi N., Zhang Y., Zhu C., Boado R.J., Pardridge W.M. (2001). Brain-specific expression of an exogenous gene after iv administration. Proc. Natl. Acad. Sci. USA.

[152] Zhang Y., Jeong Lee H., Boado R.J., Pardridge W.M. (2002). Receptor-mediated delivery of an antisense gene to human brain cancer cells. J. Gene Med. Cross-Discip. J. Res. Sci. Gene Transf. Its Clin. Appl..

[153] Mamot C., Drummond D.C., Noble C.O., Kallab V., Guo Z., Hong K., Kirpotin D.B., Park J.W. (2005). Epidermal growth factor receptor–targeted immunoliposomes significantly enhance the efficacy of multiple anticancer drugs in vivo. Cancer Res..

[154] Wu D., Yang J., Pardridge W.M. (1997). Drug targeting of a peptide radiopharmaceutical through the primate blood-brain barrier in vivo with a monoclonal antibody to the human insulin receptor. J. Clin. Investig..

[155] Schwarze S.R., Ho A., Vocero-Akbani A., Dowdy S.F. (1999). In vivo protein transduction: Delivery of a biologically active protein into the mouse. Science.

[156] Liu L., Guo K., Lu J., Venkatraman S.S., Luo D., Ng K.C., Ling E.-A., Moochhala S., Yang Y.-Y. (2008). Biologically active core/shell nanoparticles self-assembled from cholesterol-terminated PEG–TAT for drug delivery across the blood–brain barrier. Biomaterials.

[157] Kanazawa T., Taki H., Tanaka K., Takashima Y., Okada H. (2011). Cell-penetrating peptide-modified block copolymer micelles promote direct brain delivery via intranasal administration. Pharm. Res..

[158] Cai B., Lin Y., Xue X.-H., Fang L., Wang N., Wu Z.-Y. (2011). TAT-mediated delivery of neuroglobin protects against focal cerebral ischemia in mice. Exp. Neurol..

[159] Tosi G., Costantino L., Rivasi F., Ruozi B., Leo E., Vergoni A.V., Tacchi R., Bertolini A., Vandelli M.A., Forni F. (2007). Targeting the central nervous system: In vivo experiments with peptide-derivatized nanoparticles loaded with Loperamide and Rhodamine-123. J. Control. Release.

[160] Vergoni A.V., Tosi G., Tacchi R., Vandelli M.A., Bertolini A., Costantino L. (2009). Nanoparticles as drug delivery agents specific for CNS: In vivo biodistribution. Nanomed. Nanotechnol. Biol. Med..

[161] Sabatier J., Vives E., Mabrouk K., Benjouad A., Rochat H., Duval A., Hue B., Bahraoui E. (1991). Evidence for neurotoxic activity of tat from human immunodeficiency virus type 1. J. Virol..

[162] Costantino L., Gandolfi F., Tosi G., Rivasi F., Vandelli M.A., Forni F. (2005). Peptide-derivatized biodegradable nanoparticles able to cross the blood–brain barrier. J. Control. Release.

[163] Demeule M., Currie J.C., Bertrand Y., Ché C., Nguyen T., Régina A., Gabathuler R., Castaigne J.P., Béliveau R. (2008). Involvement of the low-density lipoprotein receptor-related protein in the transcytosis of the brain delivery vector Angiopep-2. J. Neurochem..

[164] Göppert T.M., Müller R.H. (2005). Polysorbate-stabilized solid lipid nanoparticles as colloidal carriers for intravenous targeting of drugs to the brain: Comparison of plasma protein adsorption patterns. J. Drug Target..

[165] Kreuter J., Ramge P., Petrov V., Hamm S., Gelperina S.E., Engelhardt B., Alyautdin R., Von Briesen H., Begley D.J. (2003). Direct evidence that polysorbate-80-coated poly (butylcyanoacrylate) nanoparticles deliver drugs to the CNS via specific mechanisms requiring prior binding of drug to the nanoparticles. Pharm. Res..

[166] Michaelis K., Hoffmann M.M., Dreis S., Herbert E., Alyautdin R.N., Michaelis M., Kreuter J., Langer K. (2006). Covalent linkage of apolipoprotein e to albumin nanoparticles strongly enhances drug transport into the brain. J. Pharmacol. Exp. Ther..

[167] Lockman P.R., Oyewumi M.O., Koziara J.M., Roder K.E., Mumper R.J., Allen D.D. (2003). Brain uptake of thiamine-coated nanoparticles. J. Control. Release.

[168] Eavarone D.A., Yu X., Bellamkonda R.V. (2000). Targeted drug delivery to C6 glioma by transferrin-coupled liposomes. J. Biomed. Mater. Res. Off. J. Soc. Biomater. Jpn. Soc. Biomater. Aust. Soc. Biomater. Korean Soc. Biomater..

[169] Visser C.C., Stevanović S., Heleen Voorwinden L., Gaillard P.J., Crommelin D.J., Danhof M., De Boer A.G. (2004). Validation of the transferrin receptor for drug targeting to brain capillary endothelial cells in vitro. J. Drug Target..

[170] Saul J.M., Annapragada A., Natarajan J.V., Bellamkonda R.V. (2003). Controlled targeting of liposomal doxorubicin via the folate receptor in vitro. J. Control. Release.

[171] Mora M., Sagristá M.-L., Trombetta D., Bonina F.P., De Pasquale A., Saija A. (2002). Design and characterization of liposomes containing long-chain N-AcylPEs for brain delivery: Penetration of liposomes incorporating GM 1 into the rat brain. Pharm. Res..

[172] Umezawa F., Eto Y. (1988). Liposome targeting to mouse brain: Mannose as a recognition marker. Biochem. Biophys. Res. Commun..

[173] Hu K., Shi Y., Jiang W., Han J., Huang S., Jiang X. (2011). Lactoferrin conjugated PEG-PLGA nanoparticles for brain delivery: Preparation, characterization and efficacy in Parkinson’s disease. Int. J. Pharm..

[174] Wagner H.J., Pilgrim C., Brandl J. (1974). Penetration and removal of horseradish peroxidase injected into the cerebrospinal fluid: Role of cerebral perivascular spaces, endothelium and microglia. Acta Neuropathol..

[175] Hemsley K.M., Hopwood J.J. (2009). Delivery of recombinant proteins via the cerebrospinal fluid as a therapy option for neurodegenerative lysosomal storage diseases. Int. J. Clin. Pharmacol. Ther..

[176] Shirley M., Perry C.M. (2014). Aripiprazole (ABILIFY MAINTENA®): A review of its use as maintenance treatment for adult patients with schizophrenia. Drugs.

[177] Bawa R., Audette G.F., Rubinstein I. (2016). Handbook of Clinical Nanomedicine: Nanoparticles, Imaging, Therapy, and Clinical Applications.

[178] Pardridge W.M. (2011). Drug transport in brain via the cerebrospinal fluid. Fluids Barriers CNS.

[179] Papisov M.I., Belov V., Belova E., Fischman A.J., Fisher R., Wright J.L., Gannon K.S., Titus J., Gagne M., Gillooly C.A. (2012). Investigation of intrathecal transport of NPT002, a prospective therapeutic based on phage M13, in nonhuman primates. Drug Deliv. Transl. Res..

[180] Bomgaars L., Geyer J.R., Franklin J., Dahl G., Park J., Winick N.J., Klenke R., Berg S.L., Blaney S.M. (2004). Phase I trial of intrathecal liposomal cytarabine in children with neoplastic meningitis. J. Clin. Oncol..

[181] Phuphanich S., Maria B., Braeckman R., Chamberlain M. (2007). A pharmacokinetic study of intra-CSF administered encapsulated cytarabine (DepoCyt) for the treatment of neoplastic meningitis in patients with leukemia, lymphoma, or solid tumors as part of a phase III study. J. Neurooncol..

[182] Megías-Vericat J.E., García-Robles A., Company-Albir M.J., Fernández-Megía M.J., Pérez-Miralles F.C., López-Briz E., Casanova B., Poveda J.L. (2017). Early experience with compassionate use of 2 hydroxypropyl-beta-cyclodextrin for Niemann-Pick type C disease: Review of initial published cases. Neurol. Sci..

[183] Camargo F., Erickson R.P., Garver W.S., Hossain G.S., Carbone P.N., Heidenreich R.A., Blanchard J. (2001). Cyclodextrins in the treatment of a mouse model of Niemann-Pick C disease. Life Sci..

[184] Ory D.S., Ottinger E.A., Farhat N.Y., King K.A., Jiang X., Weissfeld L., Berry-Kravis E., Davidson C.D., Bianconi S., Keener L.A. (2017). Intrathecal 2-hydroxypropyl-β-cyclodextrin decreases neurological disease progression in Niemann-Pick disease, type C1: A non-randomised, open-label, phase 1-2 trial. Lancet.

[185] Maarup T.J., Chen A.H., Porter F.D., Farhat N.Y., Ory D.S., Sidhu R., Jiang X., Dickson P.I. (2015). Intrathecal 2-hydroxypropyl-beta-cyclodextrin in a single patient with Niemann-Pick C1. Mol. Genet. Metab..

[186] Berry-Kravis E., Chin J., Hoffmann A., Winston A., Stoner R., LaGorio L., Friedmann K., Hernandez M., Ory D.S., Porter F.D. (2018). Long-Term Treatment of Niemann-Pick Type C1 Disease With Intrathecal 2-Hydroxypropyl-β-Cyclodextrin. Pediatr. Neurol..

[187] Matsuo M., Shraishi K., Wada K., Ishitsuka Y., Doi H., Maeda M., Mizoguchi T., Eto J., Mochinaga S., Arima H. (2014). Effects of intracerebroventricular administration of 2-hydroxypropyl-β-cyclodextrin in a patient with Niemann-Pick Type C disease. Mol. Genet. Metab. Rep..

[188] García-Robles A.A., Company-Albir M.J., Megías-Vericat J.E., Fernández-Megía M.J., Pérez-Miralles F.C., López-Briz E., Alcalá-Vicente C., Galeano I., Casanova B., Poveda J.L. (2016). Use of 2 hydroxypropyl-beta-cyclodextrin therapy in two adult Niemann Pick Type C patients. J. Neurol. Sci..

[189] Dullenkopf A., Borgeat A. (2003). Local anesthetics. Differences and similarities in the “-cains”. Anaesthesist.

[190] Covino B.G. (1986). Pharmacology of local anaesthetic agents. Br. J. Anaesth..

[191] Chahar P., Cummings K.C. (2012). Liposomal bupivacaine: A review of a new bupivacaine formulation. J. Pain Res..

[192] Boogaerts J.G., Lafont N.D., Declercq A.G., Luo H.C., Gravet E.T., Bianchi J.A., Legros F.J. (1994). Epidural administration of liposome-associated bupivacaine for the management of postsurgical pain: A first study. J. Clin. Anesth..

[193] Smoot J.D., Bergese S.D., Onel E., Williams H.T., Hedden W. (2012). The efficacy and safety of DepoFoam bupivacaine in patients undergoing bilateral, cosmetic, submuscular augmentation mammaplasty: A randomized, double-blind, active-control study. Aesthet. Surg. J..

[194] Hänggi D., Etminan N., Mayer S.A., Aldrich E.F., Diringer M.N., Schmutzhard E., Faleck H.J., Ng D., Saville B.R., Macdonald R.L. (2019). Clinical Trial Protocol: Phase 3, Multicenter, Randomized, Double-Blind, Placebo-Controlled, Parallel-Group, Efficacy, and Safety Study Comparing EG-1962 to Standard of Care Oral Nimodipine in Adults with Aneurysmal Subarachnoid Hemorrhage [NEWTON-2 (Nimodipine Microparticles to Enhance Recovery While Reducing TOxicity After SubarachNoid Hemorrhage)]. Neurocrit. Care.

[195] Hänggi D., Etminan N., Macdonald R.L., Steiger H.J., Mayer S.A., Aldrich F., Diringer M.N., Hoh B.L., Mocco J., Strange P. (2015). NEWTON: Nimodipine Microparticles to Enhance Recovery While Reducing Toxicity After Subarachnoid Hemorrhage. Neurocrit. Care.

[196] Hänggi D., Etminan N., Aldrich F., Steiger H.J., Mayer S.A., Diringer M.N., Hoh B.L., Mocco J., Faleck H.J., Macdonald R.L. (2017). Randomized, Open-Label, Phase 1/2a Study to Determine the Maximum Tolerated Dose of Intraventricular Sustained Release Nimodipine for Subarachnoid Hemorrhage (NEWTON [Nimodipine Microparticles to Enhance Recovery While Reducing Toxicity After Subarachnoid Hemorrhage]). Stroke.

[197] Etminan N., Macdonald R.L., Davis C., Burton K., Steiger H.J., Hänggi D. (2015). Intrathecal application of the nimodipine slow-release microparticle system eg-1962 for prevention of delayed cerebral ischemia and improvement of outcome after aneurysmal subarachnoid hemorrhage. Acta Neurochir. Suppl..

[198] Iliff J.J., Wang M., Liao Y., Plogg B.A., Peng W., Gundersen G.A., Benveniste H., Vates G.E., Deane R., Goldman S.A. (2012). A paravascular pathway facilitates CSF flow through the brain parenchyma and the clearance of interstitial solutes, including amyloid β. Sci. Transl. Med..

[199] Wong K.K., Liu X.L. (2012). Nanomedicine: A primer for surgeons. Pediatr. Surg. Int..

[200] Rajadhyaksha M., Boyden T., Liras J., El-Kattan A., Brodfuehrer J. (2011). Current advances in delivery of biotherapeutics across the blood-brain barrier. Curr. Drug Discov. Technol..

[201] Ozkizilcik A., Davidson P., Turgut H., Sharma H.S., Sharma A., Tian Z.R., Sharma H.S., Muresanu D.F., Sharma A. (2017). Nanocarriers as CNS Drug Delivery Systems for Enhanced Neuroprotection. Drug and Gene Delivery to the Central Nervous System for Neuroprotection: Nanotechnological Advances.

[202] Alexander A., Agrawal M., Uddin A., Siddique S., Shehata A.M., Shaker M.A., Ata Ur Rahman S., Abdul M.I.M., Shaker M.A. (2019). Recent expansions of novel strategies towards the drug targeting into the brain. Int. J. Nanomed..

[203] Bonferoni M.C., Rossi S., Sandri G., Ferrari F., Gavini E., Rassu G., Giunchedi P. (2019). Nanoemulsions for “Nose-to-Brain” Drug Delivery. Pharmaceutics.

[204] Shiekh F.A. (2014). Highlights From Recent Advances in Nanomedicine. Nanomedicine.

[205] Chakraborty S., Dhakshinamurthy G.S., Misra S.K. (2017). Tailoring of physicochemical properties of nanocarriers for effective anti-cancer applications. J. Biomed. Mater. Res. A.

[206] Shegokar R., Müller R.H. (2010). Nanocrystals: Industrially feasible multifunctional formulation technology for poorly soluble actives. Int. J. Pharm..

[207] Chan H.K., Kwok P.C. (2011). Production methods for nanodrug particles using the bottom-up approach. Adv. Drug Deliv. Rev..

[208] Naqvi S., Panghal A., Flora S.J.S. (2020). Nanotechnology: A Promising Approach for Delivery of Neuroprotective Drugs. Front. Neurosci..

[209] Guo L., Ren J., Jiang X. (2012). Perspectives on brain-targeting drug delivery systems. Curr. Pharm. Biotechnol..

[210] Vlieghe P., Khrestchatisky M. (2013). Medicinal chemistry based approaches and nanotechnology-based systems to improve CNS drug targeting and delivery. Med. Res. Rev..

[211] Cazenave J., Ale A., Bacchetta C., Rossi A.S. (2019). Nanoparticles Toxicity in Fish Models. Curr. Pharm. Des..

[212] Vishwakarma V., Samal S.S., Manoharan N. (2010). Safety and risk associated with nanoparticles-a review. J. Miner. Mater. Charact. Eng..

[213] Hagens W.I., Oomen A.G., de Jong W.H., Cassee F.R., Sips A.J. (2007). What do we (need to) know about the kinetic properties of nanoparticles in the body?. Regul. Toxicol. Pharmacol..

[214] Oberdörster G., Oberdörster E., Oberdörster J. (2005). Nanotoxicology: An emerging discipline evolving from studies of ultrafine particles. Environ. Health Perspect..

[215] Garnett M.C., Kallinteri P. (2006). Nanomedicines and nanotoxicology: Some physiological principles. Occup. Med..

[216] Yang L., Watts D.J. (2005). Particle surface characteristics may play an important role in phytotoxicity of alumina nanoparticles. Toxicol. Lett..

[217] Khan A.R., Yang X., Fu M., Zhai G. (2018). Recent progress of drug nanoformulations targeting to brain. J. Control Release.

[218] Kirchhausen T., Owen D., Harrison S.C. (2014). Molecular structure, function, and dynamics of clathrin-mediated membrane traffic. Cold Spring Harb. Perspect. Biol..

[219] Johannes L., Wunder C., Bassereau P. (2014). Bending “on the rocks”—A cocktail of biophysical modules to build endocytic pathways. Cold Spring Harb. Perspect. Biol..

[220] Merrifield C.J., Kaksonen M. (2014). Endocytic accessory factors and regulation of clathrin-mediated endocytosis. Cold Spring Harb. Perspect. Biol..

[221] Smith M.W., Gumbleton M. (2006). Endocytosis at the blood-brain barrier: From basic understanding to drug delivery strategies. J. Drug Target..

[222] Armulik A., Genové G., Mäe M., Nisancioglu M.H., Wallgard E., Niaudet C., He L., Norlin J., Lindblom P., Strittmatter K. (2010). Pericytes regulate the blood-brain barrier. Nature.

[223] Villaseñor R., Ozmen L., Messaddeq N., Grüninger F., Loetscher H., Keller A., Betsholtz C., Freskgård P.O., Collin L. (2016). Trafficking of Endogenous Immunoglobulins by Endothelial Cells at the Blood-Brain Barrier. Sci. Rep..

[224] Strazielle N., Ghersi-Egea J.F. (2013). Physiology of blood-brain interfaces in relation to brain disposition of small compounds and macromolecules. Mol. Pharm..

[225] Peters A. (1991). The fine structure of the nervous system. Neurons Their Support. Cells.

[226] Janssen S.F., van der Spek S.J., Ten Brink J.B., Essing A.H., Gorgels T.G., van der Spek P.J., Jansonius N.M., Bergen A.A. (2013). Gene expression and functional annotation of the human and mouse choroid plexus epithelium. PLoS ONE.

[227] van Deurs B., Møller M., Amtorp O. (1978). Uptake of horseradish peroxidase from CSF into the choroid plexus of the rat, with special reference to transepithelial transport. Cell Tissue Res..

[228] Balin B.J., Broadwell R.D. (1988). Transcytosis of protein through the mammalian cerebral epithelium and endothelium. I. Choroid plexus and the blood-cerebrospinal fluid barrier. J. Neurocytol..

[229] Foged C., Nielsen H.M. (2008). Cell-penetrating peptides for drug delivery across membrane barriers. Expert. Opin. Drug Deliv..

[230] Niewoehner J., Bohrmann B., Collin L., Urich E., Sade H., Maier P., Rueger P., Stracke J.O., Lau W., Tissot A.C. (2014). Increased brain penetration and potency of a therapeutic antibody using a monovalent molecular shuttle. Neuron.

[231] Sade H., Baumgartner C., Hugenmatter A., Moessner E., Freskgård P.O., Niewoehner J. (2014). A human blood-brain barrier transcytosis assay reveals antibody transcytosis influenced by pH-dependent receptor binding. PLoS ONE.

[232] Stanimirovic D., Kemmerich K., Haqqani A.S., Farrington G.K. (2014). Engineering and pharmacology of blood-brain barrier-permeable bispecific antibodies. Adv. Pharmacol..

[233] Malcor J.D., Payrot N., David M., Faucon A., Abouzid K., Jacquot G., Floquet N., Debarbieux F., Rougon G., Martinez J. (2012). Chemical optimization of new ligands of the low-density lipoprotein receptor as potential vectors for central nervous system targeting. J. Med. Chem..

[234] Xiao G., Gan L.S. (2013). Receptor-mediated endocytosis and brain delivery of therapeutic biologics. Int. J. Cell Biol..

[235] Connor J.R., Ponnuru P., Wang X.S., Patton S.M., Allen R.P., Earley C.J. (2011). Profile of altered brain iron acquisition in restless legs syndrome. Brain.

[236] Giometto B., Bozza F., Argentiero V., Gallo P., Pagni S., Piccinno M.G., Tavolato B. (1990). Transferrin receptors in rat central nervous system. An immunocytochemical study. J. Neurol. Sci..

[237] Jefferies W.A., Brandon M.R., Hunt S.V., Williams A.F., Gatter K.C., Mason D.Y. (1984). Transferrin receptor on endothelium of brain capillaries. Nature.

[238] Leitner D.F., Connor J.R. (2012). Functional roles of transferrin in the brain. Biochim. Biophys. Acta.

[239] Méndez-Gómez H.R., Galera-Prat A., Meyers C., Chen W., Singh J., Carrión-Vázquez M., Muzyczka N. (2015). Transcytosis in the blood-cerebrospinal fluid barrier of the mouse brain with an engineered receptor/ligand system. Mol. Ther. Methods Clin. Dev..

[240] Clark A.J., Davis M.E. (2015). Increased brain uptake of targeted nanoparticles by adding an acid-cleavable linkage between transferrin and the nanoparticle core. Proc. Natl. Acad. Sci. USA.

[241] Duffy K.R., Pardridge W.M. (1987). Blood-brain barrier transcytosis of insulin in developing rabbits. Brain Res..

[242] Pardridge W.M., Kang Y.S., Buciak J.L., Yang J. (1995). Human insulin receptor monoclonal antibody undergoes high affinity binding to human brain capillaries in vitro and rapid transcytosis through the blood-brain barrier in vivo in the primate. Pharm. Res..

[243] Pardridge W.M. (2007). Blood-brain barrier delivery of protein and non-viral gene therapeutics with molecular Trojan horses. J. Control Release.

[244] Baskin D.G., Brewitt B., Davidson D.A., Corp E., Paquette T., Figlewicz D.P., Lewellen T.K., Graham M.K., Woods S.G., Dorsa D.M. (1986). Quantitative autoradiographic evidence for insulin receptors in the choroid plexus of the rat brain. Diabetes.

[245] Pansky B., Hatfield J.S. (1978). Cerebral localization of insulin by immunofluorescence. Am. J. Anat..

[246] Werther G.A., Hogg A., Oldfield B.J., McKinley M.J., Figdor R., Allen A.M., Mendelsohn F.A. (1987). Localization and characterization of insulin receptors in rat brain and pituitary gland using in vitro autoradiography and computerized densitometry. Endocrinology.

[247] Marks J.L., Porte D., Stahl W.L., Baskin D.G. (1990). Localization of insulin receptor mRNA in rat brain by in situ hybridization. Endocrinology.

[248] Schwartz M.W., Bergman R.N., Kahn S.E., Taborsky G.J., Fisher L.D., Sipols A.J., Woods S.C., Steil G.M., Porte D. (1991). Evidence for entry of plasma insulin into cerebrospinal fluid through an intermediate compartment in dogs. Quantitative aspects and implications for transport. J. Clin. Investig..

[249] Wallum B.J., Taborsky G.J., Porte D., Figlewicz D.P., Jacobson L., Beard J.C., Ward W.K., Dorsa D. (1987). Cerebrospinal fluid insulin levels increase during intravenous insulin infusions in man. J. Clin. Endocrinol. Metab..

[250] Baura G.D., Foster D.M., Porte D., Kahn S.E., Bergman R.N., Cobelli C., Schwartz M.W. (1993). Saturable transport of insulin from plasma into the central nervous system of dogs in vivo. A mechanism for regulated insulin delivery to the brain. J. Clin. Investig..

[251] Manin M., Balage M., Larue-Achagiotis C., Grizard J. (1988). Chronic intracerebroventricular infusion of insulin failed to alter brain insulin-binding sites, food intake, and body weight. J. Neurochem..

[252] Dehouck B., Fenart L., Dehouck M.P., Pierce A., Torpier G., Cecchelli R. (1997). A new function for the LDL receptor: Transcytosis of LDL across the blood-brain barrier. J. Cell Biol..

[253] Matsumoto K., Chiba Y., Fujihara R., Kubo H., Sakamoto H., Ueno M. (2015). Immunohistochemical analysis of transporters related to clearance of amyloid-β peptides through blood-cerebrospinal fluid barrier in human brain. Histochem. Cell Biol..

[254] Morales C.R., Zeng J., El Alfy M., Barth J.L., Chintalapudi M.R., McCarthy R.A., Incardona J.P., Argraves W.S. (2006). Epithelial trafficking of Sonic hedgehog by megalin. J. Histochem. Cytochem..

[255] Demeule M., Poirier J., Jodoin J., Bertrand Y., Desrosiers R.R., Dagenais C., Nguyen T., Lanthier J., Gabathuler R., Kennard M. (2002). High transcytosis of melanotransferrin (P97) across the blood-brain barrier. J. Neurochem..

[256] Demeule M., Régina A., Ché C., Poirier J., Nguyen T., Gabathuler R., Castaigne J.P., Béliveau R. (2008). Identification and design of peptides as a new drug delivery system for the brain. J. Pharmacol. Exp. Ther..

[257] Shibata M., Yamada S., Kumar S.R., Calero M., Bading J., Frangione B., Holtzman D.M., Miller C.A., Strickland D.K., Ghiso J. (2000). Clearance of Alzheimer’s amyloid-ss(1–40) peptide from brain by LDL receptor-related protein-1 at the blood-brain barrier. J. Clin. Investig..

[258] Wolf B.B., Lopes M.B., VandenBerg S.R., Gonias S.L. (1992). Characterization and immunohistochemical localization of alpha 2-macroglobulin receptor (low-density lipoprotein receptor-related protein) in human brain. Am. J. Pathol..

[259] Moestrup S.K., Gliemann J., Pallesen G. (1992). Distribution of the alpha 2-macroglobulin receptor/low density lipoprotein receptor-related protein in human tissues. Cell Tissue Res..

[260] May P., Woldt E., Matz R.L., Boucher P. (2007). The LDL receptor-related protein (LRP) family: An old family of proteins with new physiological functions. Ann. Med..

[261] Marzolo M.P., Farfán P. (2011). New insights into the roles of megalin/LRP2 and the regulation of its functional expression. Biol. Res..

[262] Strazielle N., Khuth S.T., Murat A., Chalon A., Giraudon P., Belin M.F., Ghersi-Egea J.F. (2003). Pro-inflammatory cytokines modulate matrix metalloproteinase secretion and organic anion transport at the blood-cerebrospinal fluid barrier. J. Neuropathol. Exp. Neurol..

[263] Carro E., Spuch C., Trejo J.L., Antequera D., Torres-Aleman I. (2005). Choroid plexus megalin is involved in neuroprotection by serum insulin-like growth factor I. J. Neurosci..

[264] Stockinger W., Hengstschläger-Ottnad E., Novak S., Matus A., Hüttinger M., Bauer J., Lassmann H., Schneider W.J., Nimpf J. (1998). The low density lipoprotein receptor gene family. Differential expression of two alpha2-macroglobulin receptors in the brain. J. Biol. Chem..

[265] Kim D.H., Iijima H., Goto K., Sakai J., Ishii H., Kim H.J., Suzuki H., Kondo H., Saeki S., Yamamoto T. (1996). Human apolipoprotein E receptor 2. A novel lipoprotein receptor of the low density lipoprotein receptor family predominantly expressed in brain. J. Biol. Chem..

[266] Burk R.F., Hill K.E., Motley A.K., Winfrey V.P., Kurokawa S., Mitchell S.L., Zhang W. (2014). Selenoprotein P and apolipoprotein E receptor-2 interact at the blood-brain barrier and also within the brain to maintain an essential selenium pool that protects against neurodegeneration. FASEB J..

[267] Grapp M., Wrede A., Schweizer M., Hüwel S., Galla H.J., Snaidero N., Simons M., Bückers J., Low P.S., Urlaub H. (2013). Choroid plexus transcytosis and exosome shuttling deliver folate into brain parenchyma. Nat. Commun..

[268] Weitman S.D., Weinberg A.G., Coney L.R., Zurawski V.R., Jennings D.S., Kamen B.A. (1992). Cellular localization of the folate receptor: Potential role in drug toxicity and folate homeostasis. Cancer Res..

[269] Spector R. (2009). Nutrient transport systems in brain: 40 years of progress. J. Neurochem..

[270] Weitman S.D., Frazier K.M., Kamen B.A. (1994). The folate receptor in central nervous system malignancies of childhood. J. Neurooncol..

[271] Liddelow S.A., Dziegielewska K.M., Ek C.J., Johansson P.A., Potter A.M., Saunders N.R. (2009). Cellular transfer of macromolecules across the developing choroid plexus of Monodelphis domestica. Eur. J. Neurosci..

[272] Dziegielewska K.M., Habgood M.D., Møllgård K., Stagaard M., Saunders N.R. (1991). Species-specific transfer of plasma albumin from blood into different cerebrospinal fluid compartments in the fetal sheep. J. Physiol..

[273] Møllgård K., Jacobsen M., Jacobsen G.K., Clausen P.P., Saunders N.R. (1979). Immunohistochemical evidence for an intracellular localization of plasma proteins in human foetal choroid plexus and brain. Neurosci. Lett..

[274] Dziegielewska K.M., Evans C.A., Fossan G., Lorscheider F.L., Malinowska D.H., Møllgård K., Reynolds M.L., Saunders N.R., Wilkinson S. (1980). Proteins in cerebrospinal fluid and plasma of fetal sheep during development. J. Physiol..

[275] Habgood M.D., Sedgwick J.E., Dziegielewska K.M., Saunders N.R. (1992). A developmentally regulated blood-cerebrospinal fluid transfer mechanism for albumin in immature rats. J. Physiol..

[276] Dziegielewska K.M., Evans C.A., Malinowska D.H., Møllgård K., Reynolds M.L., Saunders N.R. (1980). Blood-cerebrospinal fluid transfer of plasma proteins during fetal development in the sheep. J. Physiol..

[277] Liddelow S.A., Dziegielewska K.M., VandeBerg J.L., Noor N.M., Potter A.M., Saunders N.R. (2011). Modification of protein transfer across blood/cerebrospinal fluid barrier in response to altered plasma protein composition during development. Eur. J. Neurosci..

[278] Li J., Wang H. (2023). Selective organ targeting nanoparticles: From design to clinical translation. Nanoscale Horiz..

[279] Liddelow S.A., Temple S., Møllgård K., Gehwolf R., Wagner A., Bauer H., Bauer H.C., Phoenix T.N., Dziegielewska K.M., Saunders N.R. (2012). Molecular characterisation of transport mechanisms at the developing mouse blood-CSF interface: A transcriptome approach. PLoS ONE.

[280] Ohtsuki S., Terasaki T. (2007). Contribution of carrier-mediated transport systems to the blood-brain barrier as a supporting and protecting interface for the brain; importance for CNS drug discovery and development. Pharm. Res..

[281] Qu B., Li X., Guan M., Li X., Hai L., Wu Y. (2014). Design, synthesis and biological evaluation of multivalent glucosides with high affinity as ligands for brain targeting liposomes. Eur. J. Med. Chem..

[282] Du D., Chang N., Sun S., Li M., Yu H., Liu M., Liu X., Wang G., Li H., Liu X. (2014). The role of glucose transporters in the distribution of p-aminophenyl-α-d-mannopyranoside modified liposomes within mice brain. J. Control Release.

[283] Gromnicova R., Davies H.A., Sreekanthreddy P., Romero I.A., Lund T., Roitt I.M., Phillips J.B., Male D.K. (2013). Glucose-coated gold nanoparticles transfer across human brain endothelium and enter astrocytes in vitro. PLoS ONE.

[284] Jiang X., Xin H., Ren Q., Gu J., Zhu L., Du F., Feng C., Xie Y., Sha X., Fang X. (2014). Nanoparticles of 2-deoxy-d-glucose functionalized poly(ethylene glycol)-co-poly(trimethylene carbonate) for dual-targeted drug delivery in glioma treatment. Biomaterials.

[285] Hervé F., Ghinea N., Scherrmann J.M. (2008). CNS delivery via adsorptive transcytosis. AAPS J..

[286] Bickel U., Yoshikawa T., Pardridge W.M. (2001). Delivery of peptides and proteins through the blood-brain barrier. Adv. Drug Deliv. Rev..

[287] Poduslo J.F., Ramakrishnan M., Holasek S.S., Ramirez-Alvarado M., Kandimalla K.K., Gilles E.J., Curran G.L., Wengenack T.M. (2007). In vivo targeting of antibody fragments to the nervous system for Alzheimer’s disease immunotherapy and molecular imaging of amyloid plaques. J. Neurochem..

[288] Drin G., Cottin S., Blanc E., Rees A.R., Temsamani J. (2003). Studies on the internalization mechanism of cationic cell-penetrating peptides. J. Biol. Chem..

[289] Mazel M., Clair P., Rousselle C., Vidal P., Scherrmann J.M., Mathieu D., Temsamani J. (2001). Doxorubicin-peptide conjugates overcome multidrug resistance. Anticancer Drugs.

[290] Blasberg R.G., Patlak C., Fenstermacher J.D. (1975). Intrathecal chemotherapy: Brain tissue profiles after ventriculocisternal perfusion. J. Pharmacol. Exp. Ther..

[291] Bruschi M.L. (2015). Strategies to Modify the Drug Release from Pharmaceutical Systems.

